# Bisphenol A (BPA) toxicity assessment and insights into current remediation strategies

**DOI:** 10.1039/d4ra05628k

**Published:** 2024-11-11

**Authors:** Joshua O. Ighalo, Setyo Budi Kurniawan, Banlambhabok Khongthaw, Junaidah Buhari, P. K. Chauhan, Jordana Georgin, Dison Stracke Pfingsten Franco

**Affiliations:** a Department of Chemical Engineering, Nnamdi Azikiwe University P. M. B. 5025 Awka Nigeria jo.ighalo@unizik.edu.ng; b Department of Chemical and Process Engineering, Faculty of Engineering and Built Environment, Universiti Kebangsaan Malaysia Bangi 43600 Selangor Malaysia; c Faculty of Applied Sciences and Biotechnology, Shoolini University Solan Himachal Pradesh 173229 India; d Department of Civil and Environmental, Universidad de la Costa, CUC Calle 58 # 55–66 Barranquilla Atlántico Colombia francodison@gmail.com

## Abstract

Bisphenol A (BPA) raises concerns among the scientific community as it is one of the most widely used compounds in industrial processes and a component of polycarbonate plastics and epoxy resins. In this review, we discuss the mechanism of BPA toxicity in food-grade plastics. Owing to its proliferation in the aqueous environment, we delved into the performance of various biological, physical, and chemical techniques for its remediation. Detailed mechanistic insights into these removal processes are provided. The toxic effects of BPA unravel as changes at the cellular level in the brain, which can result in learning difficulties, increased aggressiveness, hyperactivity, endocrine disorders, reduced fertility, and increased risk of dependence on illicit substances. Bacterial decomposition of BPA leads to new intermediates and products with lower toxicity. Processes such as membrane filtration, adsorption, coagulation, ozonation, and photocatalysis have also been shown to be efficient in aqueous-phase degradation. The breakdown mechanism of these processes is also discussed. The review demonstrates that high removal efficiency is usually achieved at the expense of high throughput. For the scalable application of BPA degradation technologies, removal efficiency needs to remain high at high throughput. We propose the need for process intensification using an integrated combination of these processes, which can solve multiple associated performance challenges.

## Abstract 2

To mitigate the toxic effects of BPA, there is a need to utilize hybrid remediation strategies for its removal from the aqueous environment.

## Graphical abstract

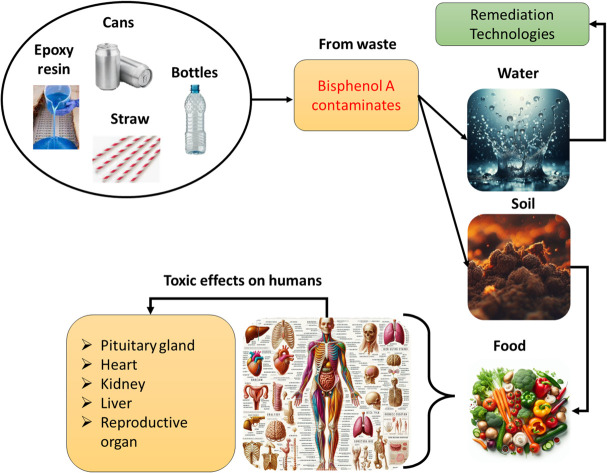

## 1. Introduction

Society's current consumption model stands out for the growing consumption of industrial compounds in processes that use epoxy resins and polycarbonate plastics, which contain bisphenol A (BPA).^1,2^ Among the products consumed around the world are beverage and food packaging, toys, and medical devices.^3,4^ These products are consumed in most cultures and social classes encompassing millions of people, resulting in an exponential increase in the release of BPA into the environment, raising concerns about its toxic effects throughout the food chain.^5^ Its chemical and physical characteristics are similar to those of hormones and can easily exhibit effects close to estrogen.^6^ Owing to this behavior, BPA can generate disturbances in the endocrine system, affecting the regulation and production of hormones.^7^

Other studies also relate its toxic effects to the formation of cancer cells, increased obesity rates, decreased fertility, and propensity for diabetes.^8^ These issues have raised interest in eliminating or reducing BPA exposure in various products consumed worldwide. The major concern is mainly linked to its presence in beverage and food packaging, which facilitates leaching into soil and water resources.^9^ Transport is favored when products are exposed to higher temperatures or even acidic compounds; in addition to this, the risk of contact is intensified with prolonged and continuous use of packaging.^10^ Owing to the damage caused by BPA, the search for new alternatives that aim to replace the product in terms of production has been enhanced and encouraged.^11^ The concern also involves the environment, considering its contamination in water and soil, which is intensified by inadequate disposal of various packaging, corroborating its leaching in landfills.^12,13^

Because of its characteristics such as transparency, robustness, and heat resistance, BPA is preferably used in the production of various food-grade plastic containers.^14^ The insecurity in using this product is its ability to move into products for human consumption, which leads to constant exposure of living organisms.^15,16^ The application of BPA in food-grade plastic packaging has been widely studied and discussed around the world.^17^ It is worth noting that although some studies affirm unlikely toxicity at low concentration levels, others state that continued exposure over the years causes serious damage, making it highly necessary to substitute new, more environmentally friendly alternatives in place of BPA in food-grade plastics.^18–21^

It was in the 1950s that the commercialization of BPA began in the United States (USA) and then spread throughout Europe.^22^ The increase in its production is currently being led by China with a growth rate of approximately 5%, while in Asia it increased by 13% in the early 2000s until 2006.^23^ In the USA alone, in the period from 2004 to 2006, around 1 million tons were generated, a number close to that generated in Western Europe.^24^ In the USA, BPA is called a toxic chemical with a large volume of use.^25,26^ It is worth noting that in poorer countries such as Africa and Latin America, these compounds do not have defined and established guidelines, even though it has been observed in studies that in countries such as Nigeria (Africa) the same was detected in several sources, highlighting the high risk of exposure in humans.^27^ In 2011, the use of 5.5 million tons of BPA was recorded worldwide.^28–31^ Inefficient treatments in treatment plants show that domestic sewage, together with manufacturing industries, are the main routes for BPA to enter the environment.^32^

Various technologies are used to remedy its presence in water and soil, and the treatments can be grouped into biological, chemical, and physical techniques. It is worth noting that all technologies have their limitations; therefore, other studies are analyzing the application of hybrid processes as a way to increase the process efficiency and reduce the operating costs. In particular, for BPA reported in the literature, one can observe studies on biological degradation (native microbes and enzymes),^33,34^ biochemical and photo-oxidation,^35–37^ membrane separation processes,^38,39^ thermal degradation^40–42^ and adsorption (graphene, zeolites, and modified carbon).^43–45^ These are among the most applicable technologies for decontaminating solutions containing BPA. It is essential to have a critical understanding of the efficiency of these processes across the varied ecospheres of the environment.

This study first analyzes the toxicity of BPA through its detection in environment-grade plastics. As a methodological process, a careful search in the Web of Science, Google Scholar, Scopus, and other databases is performed. A critical and current view is taken to describe the potential for the biodegradation of BPA by different species of bacteria. With this, the potential damage along the food chain is described. An in-depth analysis is described to elucidate the contamination and presence of BPA in food-grade plastic packaging. The influences of various storage conditions, such as pH and temperature parameters, are analyzed. To remediate BPA, an understanding of the entry routes (disposal of packaging and wastewater from industrial activities) and the transformation mechanisms is essential. Therefore, this entire process is also analyzed, corroborating the project to improve remediation technologies. Based on the proliferation of BPA in the environment, it was pertinent to review and discuss the current advances in physical, chemical, and biological processes for BPA remediation. To improve our understanding of these processes, an in-depth analysis of the mechanisms involved in BPA remediation is discussed for each process. This study aims to update the target audience on the problematic presence of BPA in food-grade plastics, to alert them to the extreme urgency of the need to develop safer and more sustainable alternatives.

## 2. Review methodology

**Fig. 1:**
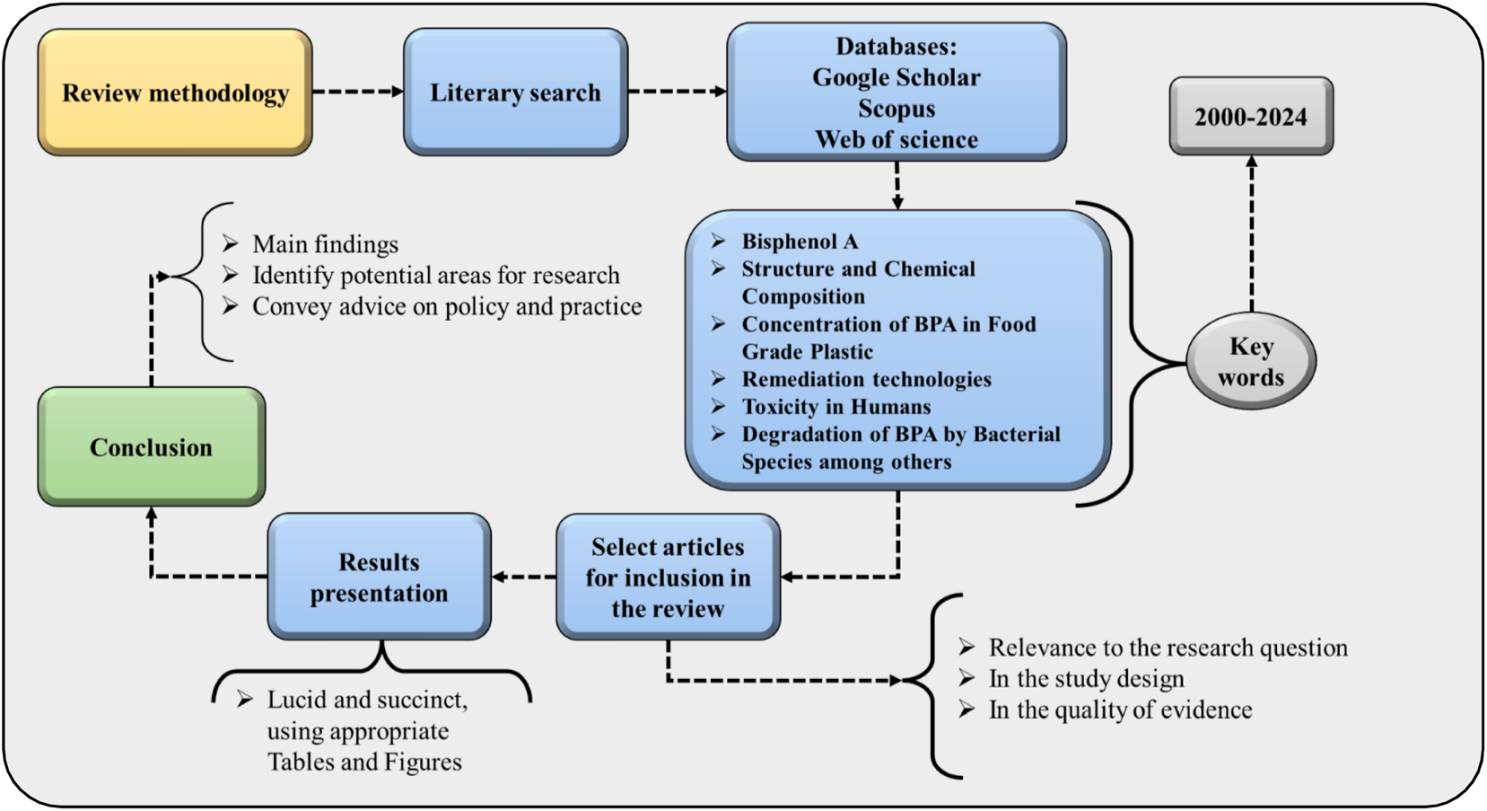
Steps carried out in the scientific methodology applied in the study.

The applied methodology is summarized in Fig. 1. Initially, a literary search was carried out in an extensive database, which includes journals present in the Web of Science, Google Scholar, Scopus, and others. Second, the words used for the search were selected, which include chemical composition and structure, BPA, its concentration in food-grade plastics, the biodegradation of BPA by strains of bacteria, the toxic effects on living organisms, and remediation technologies. The selection of articles that were published over the last 24 years was carried out, seeking to base critical analyses on current studies. For the selected studies, the relevance of the research, the quality of the evidence, and the study design were taken into consideration. The data collected were extracted in a methodical and organized manner to speed up the synthesis and analysis process. All data were carefully examined and collated to generate a comprehensive synopsis regarding the toxicity, concentration, and remediation of food-grade BPA. The results are presented succinctly and lucidly using resources such as figures and tables, explaining the main conclusions drawn. The limitations present in the literature and possible future implications for the area are also provided. Finally, the conclusion provides the main findings of the investigation to describe the main gaps existing in the political, social, and environmental spheres in the study area.

## 3. Food-grade plastics: bisphenol A concentration and sources

Characteristics such as clarity, resistance to impact and heat, and durability confirm that BPA is the most widely used synthetic compound in environmental quality plastic packaging in the world.^46^ The major concern is that once present in the environment, BPA is easily leached into groundwater and surface water, especially when exposed to acidic chemical compounds and high temperatures.^47^ The manufacturing process is one of the main sources of BPA contamination in food-grade plastics.^48^ In polycarbonate, BPA is applied as a monomer, and this process includes the polymerization of the molecules generating a more durable and robust compound.^49^ To optimize the durability and resistance, BPA is also applied as an additive in the production of epoxy resins. During these processes, the compound can also be released into the environment. The steps involved in this process are molding and polymerization. The concentration of BPA released from plastics depends on some factors such as acidity, type of plastic, temperature, the manufacturing process used, and duration of exposure time.^50^ The storage stage and application of food-grade plastic packaging also influence the sources of BPA.^51^ Since food products are packaged in plastics, depending on storage conditions, BPA can penetrate food, increasing the likelihood of exposure of humans.^52^ Another point is that many packages until they reach the final consumer are scratched or damaged, which increases the product contamination process.^53^

The recycling process is also a source of contamination in food-grade plastics, although this also has the positive aspect of reducing waste reduction.^54,55^ Residual BPA obtained at the manufacturing stage is generally present in the recycled product and can be transported to other products.^56^ The presence of BPA also occurs in other products (toys, medical and dental devices, and water bottles), which increases the sources of food-grade plastics. The intensity of contamination is high when these products are discarded inappropriately.^57^ The literature presents several reports that seek to evaluate the concentration of contaminants in food-quality packaging. The results indicate that the quantity varies depending on the pH and temperature, manufacturing process, the substance contained in the container, and the type of plastic. Several studies report its presence in various packaging (water bottles, baby bottles, and food packaging), with the highest concentrations being found in polycarbonate.^58,59^ Studies have reported the presence of BPA in breast milk samples (Asia).^60^ A study confirmed that the increase in temperature increases the transfer of BPA from the container to the food, which explains why certain samples contain higher concentrations of BPA.^61^ Canned products contain a coating that contains epoxy resins. In this investigation, it was observed that the concentration of BPA in foods highly varied, making it possible to detect low and high concentrations.^62,63^

In the literature, it is possible to observe investigations that indicate that low concentrations of BPA do not cause harm to human health; however, many claim that increasing exposure over a long period can cause serious damage, mainly due to its bioaccumulation capacity, which would increase its concentration.^64^ Due to this, an increase in research has been observed looking for more ecological and alternative materials to replace BPA in food-grade plastic containers. Some industrial processes are already manufacturing plastic packaging without the presence of BPA; among the alternative materials are stainless steel and glass.^65,66^ The great concern is not only associated with human health but also with the impact on the environment; open-air landfills, exposed to high temperatures, are one of the main sources of BPA leaching into the water table, which is intensified in countries with low income.^67^ Because of this, it is highly urgent to seek new, safer alternatives to applying BPA to food-grade plastics. The change in population behavior over the last 20 years has also changed the main sources of BPA release, as a result of which scientific advances have made it possible to determine the main transformation pathways, which are described in Table 1.

**Table 1:** Main sources of BPA release in the last 20 years and their mechanisms of action in the environment

BPA release activity into the environment	Transformation mechanism	Reference
Migration of polycarbonates	The transport of BPA into liquid foods occurs through polycarbonate *via* two routes. After the polymer hydrolysis process is catalyzed *via* hydroxide (manufacturing), residual BPA is released by diffusion into foods in contact with water and simulant. The polycondensation of BPA using phosgene (a highly toxic product) is responsible for the production of polycarbonate, which is of greater economic interest. During the hydrolysis and diffusion of polycarbonate, BPA can migrate, with hydrolysis being much more impactful in terms of release; this is due to pH values and the contact of other compounds (cations). An example was polycarbonate bottles with water stored and with a use time of seven years, when exposed to high temperatures, BPA quickly went into the water. In addition, the speed and concentration of BPA migrated will depend on the chemical composition of the solution	68
Translocation through microplastics present in water	In the manufacture of resins and polymers, BPA is classified as a common precursor, where microplastic particles manufactured with low-density polyethylene together with polycarbonate, easily release BPA into water media. Additives added to water resources (toxic organic compounds) include microplastics and intensified contamination	69
Waste management: inefficient treatments	In effluent treatment plants, BPA is leached into the hydrolytic of plastic waste. Treatment generally achieves maximum efficiency in removing endocrine disruptive compounds (*R* = 37–94%), contaminating water, soil, and air. The release occurs in the decomposition and wear of plastics. Waste paint from electronics also releases BPA due to burning computer circuit boards. Bacteria (soil/sediment) under anaerobic conditions can degrade in four steps forming tribromobisphenol A, dibromobisphenol A, and monobromobisphenol A, followed by BPA as the final product	70 and 71
Effect of temperature on plastic degradation	When polymers are subjected to high temperatures, thermal degradation occurs where BPA is synthesized into bis (4-hydroxyphenyl) methane and 4-hydroxybenzoic acid. As a result of this process, the scission of the weak bonds present in the carbonate chain and alcoholysis/hydrolysis are obtained	72
Food packaging and canned food	Food consumption is the main route of human contamination. Its contact is due to the exposure of animals and the vegetable raw materials used, therefore, bioaccumulation is due to the presence of polymers and food. Studies estimate that consumption *via* food contaminated with BPA varies from 0.48 to 1.6 g per kg body weight per day, depending on consumption habits, location, and economic and cultural background. The main routes of contamination of water and food are through epoxy resins and polycarbonates	73 and 74
Electronic waste: disposal and recycling areas	Developed and underdeveloped countries, especially the latter, are concentrated in large spaces that recycle waste from electronic products, which release large amounts of BPA. This liberation has increased in recent decades due to the constant advancement and dependence of society on electronic products. The recycling of these materials is generally done crudely by dismantling the product and separating the electronic parts (this step uses an incinerator at high temperature), the burning process releases many toxic gases into the atmosphere	75
Dentistry: use of orthodontic adhesives	Processes related to the dental area use raw materials containing high concentrations of BPA, such as orthodontic adhesives based on Bis-GMA. BPA has an estrogenic behavior that is limited to the double-ring benzoic compound. The breakdown of Bis-GMA releases BPA. Widely used in the restoration of resins, being one of the most dangerous polymers released into the environment. BPA occupies around 70% of the composition of Bis-GMA resins used in enamel restoration and dental obstructions. Its toxicity has the potential for cytotoxicity at the cellular level. Poly-BPA molecules present in the oral route can decompose into BPA, this is accelerated by pH conditions, enzymes and chemical substances present, and temperature	76
Epoxy resins	Prepolymers contain several epoxide groups and are known as epoxy resins. These resins are placed in contact with various curing agents (aliphatic amines, anhydrides, and polyamides), generating cross-linked polymers in industrial processes. Most of the resins are produced with bisphenol-A diglycidyl ether, a very low molecular weight oligomer (the main component of liquid resins)	77
Paper coins	High levels of BPA were detected in thermal paper (322 g kg^−1^). During the handling of received products, contamination can occur dermally, as BPA is sprayed on the leaves (powdery layer). When a receipt is placed next to the coin in the cash register or wallet, or even when a receipt is handled before the money, the contaminant can be transported to the paper currency	78 and 79
Use of anti-rust products	Products that contain epoxy resin are sources of BPA release, among the most common are epoxy coatings with water and high levels of dust and solids, cans that store drinks and food and are coated with protection, primers used in car bodies automobiles, electrical laminates, composites with fiber-based reinforcement, tools, and castings	80

### 3.1 Exposure to bisphenol A: toxic potential

**Fig. 2:**
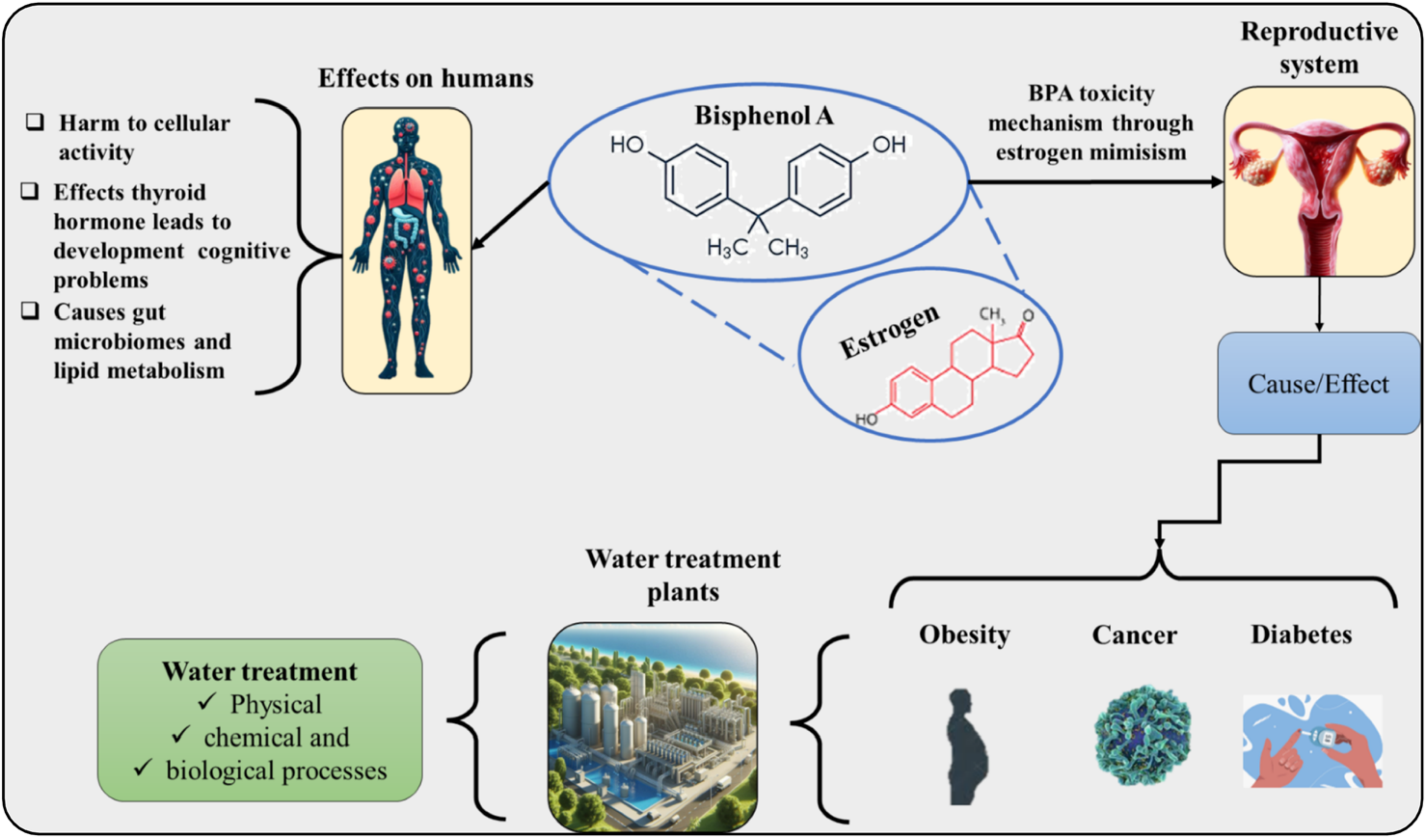
Mechanism and toxic effects of bisphenol A on human health.

The commercialization of epoxy resins and the production of polycarbonate plastics are associated with a series of health problems in humans.^81–83^ The chemical compound can imitate the estrogen hormone (Fig. 2), and hence, studies report that there may be changes in activities related to reproduction, fertility (polycystic ovary syndrome), development, increased incidence of cancer cells, and risk of obesity and diabetes.^84^ The effects on the neurological system are intensified in children interfering with learning and behavior.^85^ Children exposed to BPA in the prenatal period showed reduced bone density.^86^ Exams analyzed children's urine and observed that individuals who had a higher concentration of BPA were more aggressive and hyperactive. Women exposed to BPA in the prenatal period also had a higher rate of mental disorders, such as depression and anxiety. Studies also link the contaminant with disorders in the immune system, decreasing cellular activity and increasing the likelihood of diseases and infections.^87^ Concerning the digestive system, it was observed that even at low concentrations, BPA causes dysregulation of the intestinal barrier, reducing the microbiota and composition, which affects the immune system.^88,89^ Studies carried out on animals have shown a greater risk of neurodegenerative diseases (Alzheimer's disease, Parkinson's disease, and amyotrophic lateral sclerosis) when they are exposed to BPA, in addition to behavioral disorders and cognitive abnormalities.^90–92^ In men, a decrease in semen quality has been observed, directly affecting their fertility.^93^ Endocrine-disrupting compounds such as BPA affect endothelial function, resulting in the loss of vascular homeostasis and increasing the chances of cardiovascular diseases.^94^

The genotoxic effect has been recently observed, which is related to the pro-oxidative potential. The increase in oxidative stress is linked to the increase in the production of species that react with oxygen, the accumulation of oxidation compounds (biomacromolecules), and the change in the activities of antioxidant enzymes.^95^ This behavior is possible to occur in negative and positive estrogen receptor organisms. Once intracellular oxidation occurs, the chances of DNA damage (double- and single-strand breaks and oxidative damage) increase. Another study describes that BPA, when it also uses non-canonical estrogen as a receptor, is connected to a membrane and the receptor is coupled to the G protein. Therefore, BPA can modulate transcription factors and ion channels, which generates damage such as mitochondrial stress, damage to the endoplasmic reticulum, and damage to the amyloid polypeptide of human islets.^96^ This causes the activation of NFκB in β cells located in the pancreas, generating unregulated glucagon secretion.^96^ There are conflicting studies regarding the incidence of asthma in children due to BPA, where three studies show a higher risk of asthma in childhood, and only one reported a low risk.^97^

Damage is also caused to animals, and in the literature, it is possible to observe studies that analyze the damage caused to the reproductive system of boars and sows and relate it to the levels of oxidative stress in the offspring, the quality of the meat, and muscle metabolism.^98^ Most studies are related to damage at the level of reproduction (endocrine disruption); however, there is a greater need for analysis regarding the maturation of oocytes and the potential and mechanism of their toxicity in farm animals such as cows and nuts. As in humans, mice that were exposed to BPA showed a reduction in the intestinal microbial composition and richness, where they present distinct metabolic pathways, and thus, the study highlights intestinal toxicity in vertebrates.^99^ In aquatic organisms, the effects are similar to those generated in humans, and include the following: decreased life expectancy and alteration of fat metabolism,^100^ alteration of fertility,^101^ negative regulation of ecdysone genotoxicity,^102^ ecdysone upregulation,^103^ endocrine-disrupting cytotoxicity,^104^ neurodevelopmental toxicity,^105^ alteration of retinal morphology due to alteration in thyroid hormone levels,^106^ reproductive alteration genotoxicity,^107^ modulation of antioxidant enzymes and decreased filtering capacity,^108^ and physiological and reproductive changes in fish.^109^

In plants, studies are more limited, however, it is described that BPA generates stress, which produces a large number of species that react with oxygen.^110^ These new species act as signal messengers and provoke various stress responses in plant antioxidant cells.^111^ The literature describes that BPA can imitate the cytokine hormone in plants, in addition to the role of ethylene in the oxidative stress process generated by BPA.^112^ It is also possible to observe studies that describe different changes at both the biochemical and physiological levels in plant cells.^87^ Generally, biochemical reactions correspond to changes in enzymes at temperatures close to the environment. More studies must be carried out to optimize biomonitoring and describe what the risk situations are. Therefore, plant species that are truly sensitive to different concentrations of BPA must be selected, inserted in a natural habitat, and identified biological changes in different phases of development. This will allow us to obtain answers about the relationship between the BPA dose and the associated effects, corroborating the risk assessments. Due to all these damages, there is still considerable discussion regarding the possible effects generated at low concentrations and long periods of exposure, which strengthens the urgency in finding alternative products for BPA.^113,114^

### 3.2 A case study of the fish microbiome and the presence of BPA

Due to the numbers that prove the high human exposure to BPA, the vast majority of studies use animal and human experimental models.^115,116^ However, it is observed that water compartments can present high concentrations, and this includes both saltwater and freshwater environments. This fact corroborates the detection of BPA in the tissues of fish species in both cases. An example is in the United Kingdom where, due to the disposal of wastewater, the presence of BPA ranging from 37 to 462 ng L^−1^ was detected in river samples.^117^ This is not an isolated case, and in the Mediterranean region, the concentrations can reach 30 μg L^−1^, with a minimum of 4.5 μg L^−1^.^118^ The same authors analyzed samples on the northeast coast of the Mediterranean, where a minimum concentration of 8.85 and a maximum of 14.76 μg L^−1^ (Sea of Marmara and Black Sea) were observed. The authors highlight that the variation is influenced by meteorological variables that correspond to the seasons. Research highlights the influence of the presence of BPA on aquatic biota and its exposure to certain species. The fish species *Dicentrarchus labrax*, *Trachurus*, and *Scomber colias* were analyzed in the northeastern region of the Atlantic Ocean, and both fish tissues showed high levels of BPA proportional to the ingestion of microplastics.^119^ As a result, several investigations describe that microplastics are one of the main sources of BPA contamination in aquatic environments.^120^

It is also described that the microbiome of most fish species is potentially sensitive to BPA contamination. Most reports that analyze the impacts of the contaminant on fish are on a laboratory scale using zebrafish as an experimental model. These studies observed that, just as it occurs in mammals, BPA changes the chemical composition and generates behavioral disorders in the living organism.^121^ Different genera of zebrafish were exposed for 90 days to a concentration of up to 2 μg L^−1^ of BPA, resulting in a reduction in the pathogenic genus *Hyphomicrobium* and a significant increase in *Lawsonia*.^122^ An impoverishment of the microbiota of the intestinal tract was also reported after the presence of BPA; in contrast, in the host model, a response to oxidative injuries and inflammation was observed. Zebra fish of both sexes were analyzed based on their response to the microbiome in the presence of BPA and its other replacement products, 10 days-old larvae showed a decrease in microbial diversity, especially at higher concentrations.^123^ BPA derivatives showed diverse effects on the microbiome community. Finally, despite the composition of the microbiota having a behavior opposite to host toxicity, no noticeable effect was observed on the behavior of the larvae.^124^ A concentration of 2000 μg L^−1^ was added in contact with adult zebrafish for 35 days. In the end, it was reported that the fish showed an increase in muscle triglycerides and gastrointestinal dysbiosis.^125^ These studies corroborate the elucidation of how BPA affects the gastrointestinal microbiota of aquatic biota, serving as a support for the comparison of investigations with mammals.^126,127^

## 4. Mechanisms of BPA toxicity in food-grade plastics

The scientific community has continuously studied BPA for two reasons: the first is due to its wide use in the most varied industrial processes, and the second is its ability to contaminate food and drinks, generating toxic effects on animals, humans, and plants. The first point is that BPA is considered an endocrine disruptor due to its ability to imitate estrogen. This hormone is fundamental for the functioning of the body's organs and tissues, in addition to being important for the development in the early stages of growth.^128,129^ Once acting like estrogen, BPA alters the functioning of the endocrine system, generating changes in the activity and hormonal production of the human body. As already mentioned, this action generates several problems, among the most common of which are changes in fertility, diabetes, obesity, and cancer.^130^ At a cellular level, it alters the cell activities by generating oxidative stress, which can cause damage to membranes, chemical composition, and even ADN.^131^ Its toxic potential can occur in different signaling pathways in the human body. One example is the disturbance of enzymatic activities involving lipid metabolism, which causes disorders resulting in problems such as diabetes and obesity.^132–134^ One of the specific receptors present in the human body that BPA can interfere with is that of the thyroid hormone, this causes the interruption of signaling, generating health problems, including behavioral and developmental changes.

A study^135^ showed that higher levels of thyroid hormone were associated with contact *via* breast milk in newborns, being more evident in female children. Another point is its change in the activities of microorganisms present in the intestinal tract and digestive system, which causes changes and a possible decrease in the immune system, in addition to altering the dynamics of metabolism and other physiological processes that are involved.^91^ Therefore, BPA damage can occur through various mechanisms, and its ability to bind to estrogen receptors is the most discussed in the literature, as a result of which changes in cellular functions and microbial activity of the intestinal system are observed, intensified by direct effects on signaling pathways. This entire process causes considerable damage to health, which corroborates the intense efforts to develop new, more sustainable, and safer alternatives to the BPA present in various consumer products. Therefore, a reduction in exposure routes is achieved, reducing damage throughout the food chain.^136,137^

### 4.1 Regulatory guidelines and limits for BPA exposure

Due to the serious damage and constant threat of BPA to human health, guidelines have been set and regulatory limits established in some countries. To achieve this, scientific studies were used to protect the society from the potentially harmful effects of the contaminant. The Environmental Protection Agency (APA), located in the United States, decreed 50 μg per kilogram of body weight per day as a reference dose, and this would be the maximum level of exposure considered non-harmful.^138^ The US Federal Drug Administration also decreed guidelines that limit the exposure to contaminants in products for children's use, such as formula packaging and baby bottles. The guidelines established by the European Union were set by the European Food Safety Authority, where a daily amount of 4 μg per kilogram of body weight per day is allowed. This daily dose indicates the limit that can be exposed without causing harm to health.^139^ Unlike American legislation, the European Union does not allow the use of the contaminant in cups, straws, and baby bottles. Canada has defined a maximum concentration of up to 0.005 mg of BPA present in water, which is acceptable; however, Health Canada has warned the population about the presence of BPA in baby bottles and baby formula packaging, warning the preference for smaller packaging sustainable. Countries such as Australia, Japan, and China have created guidelines with tolerance limits for BPA in different environments and packaging. Despite public policies that seek to limit contact with BPA, stricter measures are still needed, especially in less developed countries. Together, debates are needed to confirm whether these limits are truly safe for long-term exposure. Therefore, continued research is recommended to obtain a broader understanding of the real potential health risks together with new, more viable, and safer alternatives to BPA.^140,141^

The presence of BPA in liquids such as beverages generally occurs in products that feature the coating of bottle caps, cans, and other pipes that line the entire water supply.^142^ As shown in Table 2, when compared to other solid foods, the presence of BPA in beverages is much less reported and at lower concentrations. Two factors can justify this: first the LODs are high in the most varied analytical methods used, and second, the concentration of coating used in beverage cans is lower than that used in solid foods. BPA has never been observed in liquids packaged in glass; however, it has been detected in plastic packaging and other types of canned goods. BPA has been detected in mineral water packaged with polyethylene terephthalate.^144^ The highly low concentrations suggest that the main sources of BPA are bottle closures.^162^ The presence of BPA in dairy products can also occur during production due to contact with equipment and other utensils used during food processing. Ruminant feed can also generate BPA contamination, because it contains alkphenols, such as octylphenol and nonylphenol.^163^ BPA and its derivatives are present in milk, which are presented in Table 2; in this case, the packaging may play a minor role in contamination. This is because variables such as heat treatments and the various utensils used in production can lead to greater contamination. Fatty dairy products that are secreted by cows can have a high presence of BPA. Few studies report the release of BPA throughout the dairy product production chain, and more studies are needed in this regard. In this same sense, more studies are needed regarding BPA in non-canned fruits or vegetables. In the case of canned products, it is possible to observe a considerable and precise estimate regarding their content, as shown in Table 2.^155,159,164^ In general, canned vegetables constitute a highly heterogeneous group of foods (tomatoes, lentils, and mushrooms), together with water, other preservatives, and even oil in some cases. The variation in these products may reflect a high variation in the concentration of BPA. An earlier study^157^ observed that people who consume canned vegetables in greater quantities have higher levels of canned vegetables in their urine. Likewise, the presence of BPA in meat occurs due to the migration of the packaging with the solid, and this can be intensified due to the production methods and the animal's diet. Table 2 also describes BPA levels in cereals, which can be detected in plastic packaging and cans; however, more studies must be conducted for these foods.

**Table 2:** Presence of BPA in various human consumption products

Subcategory	LOG (ng g^−1^)	LOQ (ng g^−1^)	Min–max (μg kg^−1^)	Country and reference
Energy and soft cola, drinks, cola, juice, beer	0.02	0.02–8.1	—	Belgium^143^
Water	0.00075	0.00073–0.102	—	Italy^144^
Soft drinks	0.18	0.54	0.54–4.98	Italy^145^
Soft drinks	0.10	0.40	0.4–10.2	Greece^146^
Milk	0.15	—	0.99–2.64	Spain ^147^
Milk	0.30–4.20	1–14.00	<1–521	Italy^148^
Cheese spreads, milk, brown cheese, and hard cheese	—	0.10	<0.10	Norway^149^
Milk	3	9	3–169	Italy^150^
Soft, energy, and beer	0.1–9.3	—	0.1–3.4	Austria^151^
Beans, haricots, red kidney beans, young peas, crushed tomatoes and lentils	1.1–7.4	—	8.5–35	Austria^152^
Tomato	15.4–20	51.3–66.9	20.5–115.3	Italy^153^
Carrots, peas, bean shoots, artichokes, and mixed vegetables	0.016	0.55	8.90–304	Spain ^154^
Green beans, red pepper, asparagus and lentils mushroom	0.3–1.1	0.9–3.5	7.1–959	Spain ^155^
Tomato	0.09	0.26	0.31–235.88	Italy^156^
Tomato paste and mushrooms	0.60	1.9	4.9–66	Greece^146^
Pineapple and peaches	0.3–1.1	0.9–3.5	6.1–13	Spain ^155^
Meat	—	2–4	6.9–13	Sweden^157^
Tripe meat balls	0.30–1.10	0.90–3.50	9.2–341	Spain ^155^
Poultry, meat, offals and game, delicatessen meats	0.02	1.2	0.09–60.19	France^158^
Ravioli, corn	—	0.1	0.94–73.1	Belgium^143^
Pasta, bread, burns, flour, and breakfast cereals	—	—	0.1–0.24	Norway^149^
Eggs, oil, products, butter and margarine	0.21	—	0.045–4.51	France^159^
Honey	270	800	—	Italy^160^
Mustard seeds	10	30	10–8350	Switzerland^161^
Sauces	0.6	1.9	—	Greece^146^

## 5. Bacterial degradation of bisphenol A

Several studies have been developed, mainly in the last decade, seeking to optimize the BPA decontamination process mainly in water compartments and the soil. An alternative is the application of colonies of different species of bacteria that have the potential to degrade the chemical compound.^165^Table 3 describes the variety of species and strains analyzed and their degradation potential. The study by Sasaki *et al.* (2005), used the species *Sphingomonas* sp. strain AO1 isolated in a WTP (water treatment plant) located in Asia (Japan). Degradation was efficient since the microorganism used the chemical compound as a carbon source, where intermediates were generated, with a lower toxic potential. The system showed 100% removal for a concentration of 115 μg mL^−1^. Another study isolated the species *Sphingobium* sp. BiD32 from sediment and soil samples, the consumption of the chemical compound as the only source of energy allowed degradation through a sequence of chemical reactions, generating water and CO_2_ (carbon dioxide) as final products.^184^ Similar degradation results were also obtained with other species such as *Rhodococcus* sp. and *Pseudomonas putida*^185^ and *Arthrobacter* sp.^186^ The efficiency of the process at a cost-benefit level depends on the operating conditions of the environment; therefore, the control and manipulation of variables such as pH, temperature, and quantity of nutrients and water is essential. Despite the positive results and many challenges to be overcome, a limitation is ensuring optimal degradation even in the presence of other chemical compounds. Another aspect is the scale of the process, since under real environmental conditions, it requires a large number of bacteria, coupled with the difficult control of environmental variables, which can limit the proliferation of colonies, affecting degradation.

**Table 3:** Biodegradation of BPA in food-grade plastics by bacteria

Bacterial strains	Concentration	Bisphenol A removal (%)	Reference
KU-3 bacterial strains (Chennai coastal region, India)	250–1000 ppm	74	166
K-6 bacterial strains (Chennai coastal region, India)	250–1000 ppm	78	167
KU-8 bacterial strains (Chennai coastal region, India)	250–1000 ppm	81	167
*Aeromonas hydrophila*	60–120 mg L^−1^	79	168
*Chlorella vulgaris*	20 mg L^−1^	>50	169
*Sphingomonas* sp. strain AO1	115 μg mL^−1^	100	170
*Bacillus* sp. GZB	10 mg L^−1^	100	171
*Bacillus pumilus*	10 ppm	100	172
*Pseudomonas putida* YC-AE1	1000 mg L^−1^	93	173
*Bacillus* sp.GZ	100 mg L^−1^	93	174
*Pseudomonas putida* KT2440	—	46	175
*Pseudomonas* sp. LBC1	160 μM	100	176
Bacterial consortium	50 mg L^−1^	90	177
*Sphingobium* sp. BiD32	2 mg L^−1^	10	178
*Pseudoxanthomonas* sp. strain NyZ600	50 mg L^−1^	45	179
*Sphingomonadaceae*	5000 μg L^−1^	99	180
*Bacillus* sp. AM1	25 ppb	84	181
*Dracaena sanderiana*	100 mg L^−1^	99	182
*Sphingomonas* sp. strain AO1	115 μg mL^−1^	100	170
*Chlorella pyrenoidosa*	30 mg L^−1^	67	183

### 5.1 Degradation mechanism

The process of degradation of the BPA molecule by bacteria involves several routes, which allow the molecule to be broken down into less toxic and simpler compounds. These routes encompass non-enzymatic and enzymatic processes and include a diversity of colony species. The production of BPA hydrolase enzymes is one of the main mechanisms and consists of the ability of these enzymes to break the bonds that unite the BPA molecule.^187^ Hydrolases are produced by bacteria that develop when using BPA as a source of energy and carbon. These enzymes were found in several colonies (*Sphingobium japonicum*, *Pseudomonas putida*, and *Rhodococcus* sp.) that degraded the compound efficiently.^188–190^ Non-enzymatic processes are also observed, among which biotransformation and adsorption stand out. In the case of adsorption, the interaction of BPA (adsorbate) molecules occurs, which are attracted *via* physical interactions to the surface of the bacteria cells, followed by absorption and degradation. Biotransformation is related to chemical changes that occur in the contaminant through the activity of enzymes produced by bacteria, and the products generated in the end are less complex and less toxic.^191^ In this process, metabolic pathways are formed that enable the use of the compound as a source of nutrients. In the literature, the species *Pseudomonas putida* uses BPA as the sole source of energy and carbon; however, the species *Sphingobium japonicum* consumes the compound as part of a broader energy and carbon system.^192,193^

The degradation of BPA and the mechanisms used by bacteria may vary depending on the species used and the environmental conditions (pH, temperature, humidity, *etc.*) in which they were found. In general, the entire mechanism occurs through a combination of non-enzymatic and enzymatic processes, in which BPA bonds are broken.^194,195^ Achieving total control over the mechanisms that involve degradation is fundamental in planning new strategies that aim to remedy and mitigate the environmental damage generated by the release of BPA. With the correct species and the mechanisms that the process involves, researchers can achieve maximum efficiency and the best cost-benefit in applying the method.^196^

Owing to the increasing contamination and exposure to compounds containing BPA, there is a growing need and urgency for remediation technologies such as bacterial degradation.^197,198^ The colonies of bacteria with the potential to degrade BPA into less toxic compounds have been reported in diverse environments including sediments, treatment plants, and soil samples. Once new intermediate compounds are formed, they can be reused or discarded in safe places.^199^ With these practices, it is possible to reduce the volume of waste from plastics and microplastics, corroborating with less exposure, mainly to less toxic chemical molecules. An area of research that encompasses degradation by bacteria and the management of solid waste is the use of bioreactors, and these structures provide the best environmental conditions for rapid colony growth through accelerated metabolism.^200^ Bioreactors can isolate cultures where BPA is then added for subsequent degradation.^201^ Another natural process is composting. These cases also make use of microorganisms that decompose organic material (garden waste and fruit and vegetable waste). This technique also makes it possible to break down BPA molecules, supporting the management of plastic waste and consequently reducing the environmental impact and mitigating harmful effects on humans, plants, animals, and aquatic organisms.^202^

These processes still need to overcome gaps such as, for example, isolating a certain species or colony capable of degrading several chemical compounds as well as its identification. This is the first step towards achieving ideal conditions for rapid metabolism and the application of a practical and cost-effective method that can be applied to large-volume BPA waste management.^203^ To overcome these barriers, degradation with bacteria has great potential in the application of bioreactor development, including the possibility of composting. These are still possible alternatives and examples of sustainable activities in combating contamination by solid waste.

## 6. Overview of technologies for the remediation of BPA-contaminated water

### 6.1 Physical processes

Physical treatment processes such as adsorption, membrane filtration, and electrocoagulation are commonly used to remove BPA contaminants from water and wastewater due to their flexibility, simplicity, and effectiveness. However, methods other than adsorption have drawbacks such as high energy and maintenance costs, lower removal capabilities, and increased risk of membrane fouling.^204^ Coagulation or flocculation treatment, a physicochemical process, is employed to remove BPA that attaches to sludge during the primary treatment stage followed by the secondary treatment stage in wastewater treatment.^205^ However, the effectiveness of these methods is limited, and its highest recorded removal record rate is 1%. In the secondary treatment stage, BPA is subjected to advanced processes, including adsorption, oxidation, advanced oxidation, and membrane technologies which have high removal efficiency. Among the water treatment processes, coagulation or flocculation is the simplest technology with low installation costs.^206^ Over the past decade, adsorption has emerged as the most widely used method for removing BPA. The raw materials for adsorbents, such as graphene, activated carbons, zeolites, and agricultural wastes, are diverse, inexpensive, and easy to maintain. These materials have proven to be promising for large-scale BPA removal applications.^207^ Managing adsorbents effectively is essential for the efficient operation of the adsorption process. This includes selection, storage, regeneration, and treatment. Such comprehensive management significantly affects the absorbent's service life, reduces costs, and improves the overall efficiency.^208^ Successfully employing adsorption methods for BPA removal requires thorough technical preparation. This includes smooth operation of equipment and diligent monitoring of parameters such as adsorbent saturation, temperature, and flow rate. Continuous assessment ensures efficient BPA removal.^209^ Advancements in adsorption techniques promise cost-effective BPA removal. Successful implementation depends on innovation in materials/methods and meticulous management/monitoring throughout the process.

#### 6.1.1 Membrane processes

Membrane separation processes (MSPs) are advanced treatments gaining popularity in water and sewage treatment plants for achieving high water purity levels and facilitating water reuse. These processes encompass two primary mechanisms: sieving and adsorption.^210^ Since the 1990s, membrane filtration has gained a lot of interest as a drinking water treatment technology due to its ability to achieve high throughput in a continuous operation mode. There are four main types of the process: reverse osmosis (RO), nanofiltration (NF), ultrafiltration (UF), and microfiltration (MF).^211^ NF and RO are usually utilized in removing organic carbon, dissolved solids, and inorganic ions. They can also be used in removing EDCS and micropollutants.^212^ They are operated at high pressures when compared to MF and UF membranes.^213^ As much as 60 million m^3^ per day of water is being treated by RO, while MF and UF have capacities of 20 million m^3^ per day, accounting for 60% of total drinking water production.^214^ UF is more popular than the other types of membrane separation technologies because it is cheaper, and can handle particles, macromolecules, suspended solids, colloids, bacteria, and viruses.^215^ Both NF and RO membranes can effectively remove various types of micropollutants (MPs) not only *via* charge effects and adsorption but also through size exclusion.^216^ Various types of membranes employed in treatments of BPA are presented in Table 4.

**Table 4:** Various membrane technologies and their removal efficiencies^a^

Membrane	Type	Parameter condition	Remove efficiency	Reference
Layer-by-layer (LBL) biocatalytic	NF	85 cm^2^; pH 6; operation pressure, 2 bar; temperature, 25 ± 0.5 °C, salt concentration, 1000 ppm; crossflow velocity, 0.2 m s^−1^	92.5%	(Li *et al.*, 2020)^223^
Polyamide NF membrane	NF	2–3 cm (water level), 25 °C, pH = 7–9.7, 200 rpm for 2 min	88.5%	(Wang *et al.*, 2020)^224^
Dynamic electrodeposited CuO/carbon membrane (DECuO/CM)	MF	RhB concentration 300 mg L^−1^, voltage 2.5 V, electrode distant 5.75 cm, flow rate 1.2 mL min^−1^, electrolyte (Na_2_SO_4_) concentration 5 g L^−1^, and feed pH 5.3	98.04%	(Li *et al.*, 2020)^225^
PVDF_MW_ catalytic-membrane	MF	12 cm^2^, pH: 7.0, 531.6 eV	40%	217
PVC membrane	UF	35 cm^2^, 300 mL, 100 kPa for 10 min	60%	218
PES-SiO_2_ hollow fibre membranes	UF	1 bar, pH: 7, feed concentration: 10 and 100 μL^−1^	95%	211
Forward osmosis membrane	Forward osmosis	15 bar, permeate flow of 1.6 mL min^−1^, concentrate flow of 80 mL min^−1^ and a RO flux of 7 L m^−2^ h^−1^	40%	219
TW30 membrane	RO	15 bar, 10 μg L^−1^, pH: 7	84%	220
TW30-4040, polyamide	RO	NDA	90%	221

a NDA: no data available.

MF (0.2 μm) and UF (0.05 μm) may retain BPA by the interaction of the chemical with particulates, as the particulate's size is larger than the pore sizes of MF and UF membranes.^222^ Up to 92.5% BPA removal efficiency was achieved due to the combined effect of laccase catalysis, membrane rejection, and adsorption.^223^ This performance was obtained under optimal conditions of 2 bar pressure, competitive laccase loading (238.8 ± 3.5 μg cm^−2^), and laccase activity (0.6 U cm^−2^) (Li *et al.*, 2020).^223^ Wang *et al.*, (2020)^224^ proved that in the treatment of Songhua river water spiked with micropollutants, the percentage removal of BPA significantly increased to 88.5%, surpassing the efficiency of single NF without coagulation, which achieved only 60.7% removal. After the fourth usage cycle, Li *et al.*, (2020)^225^ observed a remarkable 99.8% removal efficiency for BPA using combined electrocatalytic oxidation and MF. The DECuO/CM membrane, featuring a permeability of 823.03 L (m^2^ h bar)^−1^, was fabricated by depositing CuO onto the carbon membrane. The integration of PAA/PVDF membranes with the catalytic process using nZVI/H_2_O_2_ under optimal conditions (50 L m^−2^ h^−1^, 10 mM of H_2_O_2_) led to a notable 80% improvement in BPA treatment efficiency.^217^

UF is achieved through pores ranging between 5 and 20 nm in size, with molecular weight cutoffs of 1000 to 100 000 Da.^226^ In both MF and UF processes, higher BPA concentrations in the feed solution led to increased adsorption efficiency. The estimated BPA removal rates were 36–72% for MF and 45–67% for UF.^227^ To enhance the membrane performance, researchers have incorporated inorganic materials during membrane fabrication alongside MWCNTs and PVC-II. Under optimal conditions of 35 cm^2^, 300 mL, and 100 kPa for 10 minutes, BPA degradation reached 60%. Additionally, the researcher noted that the retention of BPA decreases with the increase in pressure.^218^ Tian *et al.*, (2021)^228^ developed a piezoelectric membrane using SnS2 nanosheet-coated carbon nanofibers (CNFs) to generate hydroxyl free radicals for BPA degradation under ultrasound. They found the highest BPA adsorption with the UF membrane, followed by NF and RO membranes. The efficient reduction of BPA degradation was achieved with 97% efficiency within 80 minutes, respectively, through piezocatalysis of 0.5-SnS2/CNFs under ultrasonication in the darkness. However, Muhamad *et al.*, (2016b) demonstrated that the UF membrane system effectively removes BPA, with the membrane surface containing SiO_2_ nanoparticles and hydroxyl bonding groups of BPA. The variations in feed pH notably influenced BPA elimination, achieving the highest rejection (90%) at pH 7 and the lowest removal (20%) at pH 10. The optimization process of the model identified the following optimum conditions for BPA removal: 1 bar pressure, pH 6.7, 10 μg L^−1^ BPA concentration, and 10 minutes of filtration, resulting in 99.61% removal efficiency. Data from the RSM design confirmed that BPA removal under these optimum conditions was consistent at 99.61%, aligning with experimental values obtained in the UF membrane system.^229^

RO, primarily used for desalination due to its effective particulate rejection capabilities, has been suggested as an additional step in sewage treatment. Multiple research articles have identified RO as the most promising and efficient method for removing organic micropollutants (OMPs), including BPA.^230^ In terms of forward osmosis rejection efficiency for BPA, the polyamide thin-film composite membrane (PA-TFC) achieved 25% removal, while the polysulfone (PSf) membrane substrate demonstrated 91% removal from the feed solution.^231^ The concentrate produced from municipal wastewater recycling processes, known as reverse osmosis concentrate (ROC), presents notable environmental and health hazards due to its elevated levels of harmful compounds, such as phenolic chemicals like BPA. Under optimal conditions of 25 °C, a [Fe(vi)]/[BPA] ratio of 50, and an initial pH of 8.0 in the ROC, BPA degradation surpassed 90% after a 90 minutes reaction period.^232^ However, Moreira *et al.* (2019a) reported BPA removal from water *via* RO membrane separation. The RO membrane showed superior efficiency in removing BPA from samples containing 10 μg L^−1^ at 15 bar pressure, and the removal efficiency rate was 84%.

#### 6.1.2 Adsorption

Adsorption is the transfer of adsorbate from the liquid or gas phase to the surface or interface of the adsorbent, representing a surface phenomenon. Adsorption serves as a primary mechanism facilitating the removal of BPA through membrane filtration, along with size exclusion and charge repulsion. This process is anticipated to be influenced by hydrophobic interactions and hydrogen bonding. Notably, adsorption has demonstrated efficacy owing to its affordability, simplicity, environmentally friendly operation, the availability of various adsorbents, and the potential for renewal and reusability.^233^ The adsorption rate depends on adsorbent properties, adsorbate nature, and solution conditions. Factors affecting adsorption include temperature, experimental parameters (*e.g.*, time, pH), characteristics of adsorbate and adsorbent, and the presence of other pollutants.^234^ Researchers utilize various adsorbents such as zeolites, clay minerals, nanomaterials, graphene, activated carbons, imprinted polymers, biopolymers, and agricultural wastes for removing BPA from water. The effectiveness depends on their adsorption capacity.^235^ Adsorbents are grouped into natural ones (*e.g.*, chitosan, clays), activated carbons, graphene-based materials, nanomaterials, composite materials, imprinted polymers, agricultural wastes, hybrid particles, and inorganic-organic-modified bentonite. However, challenges including difficulty in separating very small-sized materials from the treated matrix remain.^236^

The performance of various adsorbents for BPA uptake is summarized in Table 5. The ρ-phenylenediamine-functionalized magnetic graphene oxide (PPD-MGO) nanocomposite nanomaterial exhibited good adsorption ability for BPA and good re-usability. Under optimum conditions of 45 °C and pH 7, the maximum adsorption capacity reached 155.0 mg g^−1^. The removal rate was 99.2% after three times of adsorption with the new nanomaterials.^251^ Under Langmuir models, self-flocculated powdered activated carbon (PAC-PNIPAM) exhibited the maximum adsorption capacity of BPA by 247.532 and 116.298 mg g^−1^ when conducted at 25 °C and 40 °C, respectively.^252^ Chen *et al.* (2015) reported that the maximum adsorption capacities of BPA by the Langmuir model were 182 mg g^−1^ at pH 5.0 and 196 mg g^−1^ at pH 7.0, respectively.^253^ The adsorption of BPA onto activated carbons was employed at 298 K and pH 7.0. Carbons W20 and W20N had higher adsorption capacities (382.12 and 432.34 mg g^−1^) compared to the other tested carbons. Adsorption capacity was influenced by acidic oxygen-containing groups and surface charge density.^254^ BPA removal from aqueous solutions *via* graphene adsorption showed a maximum adsorption capacity (qm) for BPA of 182 mg g^−1^ at 302.15 K, the highest reported value among carbonaceous adsorbents.^255^ The optimal operating parameters for the degradation of BPA were determined: 0.3 mL of H_2_O_2_, pH 3, and 180 min, in the presence of Fe_3_O_4_@illite. The maximum degradation capacities of 816 mg g^−1^, 364 mg g^−1^, and 113 mg g^−1^ for epoxy BPA concentrations in resin wastewater (266 mg L^−1^), synthetic wastewater (80 mg L^−1^), and Hefei City Swan Lake (25 mg L^−1^), respectively.^256^ Moreover, Hernández-Abreu *et al.* (2020)^236^ demonstrated that activated carbons (F400 and KLP) had significantly higher maximum BPA adsorption capacities (400 and 220 mg g^−1^, respectively) compared to xerogel (78 mg g^−1^). This difference was probably due to the greater microporous volume observed in F400 and KLP-activated carbons. Similarly, at an initial BPA concentration of 100 mg L^−1^, the maximum adsorption capability of 285 mg g^−1^ is 9.3 times higher than that of commercially available activated carbon adsorbents.^250^ When the BPA concentration exceeded 342 mg L^−1^, the adsorption capacity of multi-walled carbon nanotubes modified with iron oxide and manganese dioxide (MWCNTs-Fe_3_O_4_–MnO_2_) reached saturation. The static adsorption capacities were 76.75 mg g^−1^ for MWCNTs, 44.52 mg g^−1^ for MWCNTs-Fe_3_O_4_, and 132.89 mg g^−1^ for MWCNTs-Fe_3_O_4_–MnO_2_.^246^ Activated carbon (AC) efficiently removes endocrine-disrupting chemicals from water. It shows high removal rates of BPA even at low initial concentrations (1 mg L^−1^; pH: 6.5; adsorbent: 0.2 g L^−1^) and increased substantial adsorption capacity for BPA, estimated at 254.9 ± 16.2 mg g^−1^.^238^

**Table 5:** BPA treatments using various adsorbents materials

Adsorbent	Detection of BPA	Performance	Reference
Calcium alginate/activated carbon (A-AC)	FESEM, TGA, XRD, FTIR, BET	368.3 mg g^−1^	237
Activated carbon	N_2_ adsorption, desorption isotherm, FTIR, zeta potential	100%	238
Activated carbon	TEM, VSM, EDS, XRD, XPS, FTIR	100%	239
Xerogel (RFX), a chemically activated carbon from kraft lignin (KLP), commercial activated carbon (F400)	SEM, FTIR	F400 = 407 mg g^−1^, KLP = 220 mg g^−1^, xerogel = 78 mg g^−1^	236
Cellulose acetate (cigarette butt) activated carbon	FTIR, Raman, TGA, XRD, BET, SEM and XPS	364.21 mg g^−1^	240
ρ-Phenylenediamine-functionalized magnetic graphene oxide nanocomposites (PPD-MGO)	TEM, FT-IR, VSM	155.0 mg g^−1^ (99.2%)	241
Biomass-activated carbon (*Tithonia diversifolia*)	SEM and XRD	15.69 mg g^−1^	242
nZVI-chitosan	FTIR, TEM, XRD, SEM	65.16 mg g^−1^	243
Phosphonate *Halomonas* levan (PhHL)	FTIR, XPS	26.6 mg g^−1^	244
Sulfonic acid functionalized carbonaceous adsorbent (TW-SO_3_H) from tea leaves	FTIR, SEM, EDS, TGA, EDS, TGA, Raman spectroscopy, zeta potential	236.80 mg g^−1^	245
Modification of multi-walled carbon nanotube with iron oxide and manganese dioxide (MWCNTs-Fe_3_O_4_ MnO_2_)	SEM, TEM, EDX, FTIR, VSM	132.9 mg g^−1^	246
Calcium alginate/activated carbon (A-AC)	pH_PZC_, FTIR, SEM	368.3 mg g^−1^	247
Cu-BDC MOFs, Cu-BDC@GrO (graphene oxide)	XRD, TEM, SEM, EDS, Raman, FTIR, TGA, XPS, Zetasizer and ICP-OES	182 mg g^−1^	248
Magnetic vermiculite modified (MV) poly(trimesoyl chloridemelamine) (MP)	FTIR, SEM, EDX	273.67 mg g^−1^	249
Nitrogen-containing covalent organic framework (PyTTA-Dva-COF)	QM/MM, XPS	285 mg g^−1^	250

Transmission electron microscopy (TEM), Fourier transform infrared (FT-IR) spectroscopy, vibrating sample magnetometry (VSM), X-ray diffraction (XRD), X-ray photoelectron spectroscopy (XPS), thermogravimetric analysis (TGA), energy-dispersive X-ray spectroscopy (EDS), zero-point charge determination (pH_PZC_), inductively coupled plasma-optical emission spectroscopy (ICP-OES), and quantum mechanics/molecular mechanics (QM/MM) were performed.

#### 6.1.3 Coagulation/flocculation treatment

Electrocoagulation involves passing an electric current through water, generating coagulant precursors through the electrolytic oxidation of anode materials, usually aluminum or iron. This technology effectively reduces pollutant levels. The performance of various electrocoagulation systems for BPA treatment is summarized in Table 6. Kumar *et al.* (2022)^257^ reported that an electrocoagulation (EC) device equipped with aluminum blades was used to eliminate BPA, resulting in the removal of approximately 42% of the compound. Locust bean gum (LBG) was utilized for coagulation/flocculation as a pre-treatment to reduce BPA levels in leachate prior to advanced treatments. It was reported that at a pH of 7.5, an LBG dosage of 500 mg L^−1^ resulted in a reduction efficiency of 76% for BPA.^266^ In the modified coagulation sludge process, a BPA concentration of 20 mg L^−1^ underwent complete degradation within 120 minutes at room temperature, with a pH of 7, a dosage of 3 g L^−1^ for PMS, and a dosage of 0.3 g L^−1^ for MCS.^267^ According to Dolatabadi *et al.* (2021), under optimal conditions—namely, a BPA concentration of 3.25 mg L^−1^, a current density of 12.0 mA cm^−2^, a pH of 8.5, a reaction time of 23 minutes, and an electrical energy consumption of 0.308 kW h m^−3^—the maximum removal efficiency of BPA from the aqueous environment reached 98.2%.^260^ The efficacy of electrocoagulation employing iron and aluminum electrodes for eliminating phenol from aqueous surroundings was investigated. The maximum removal efficiencies of phenol were achieved at 60 V, 80 minutes, 5 mg L^−1^, 3000 μS cm^−1^, and pH levels of 5 and 7 with aluminum and iron electrodes, respectively. The removal efficiencies were reported at 94.72% for aluminum electrodes and 98.0% for iron electrodes, respectively.^268,269^ Ambauen *et al.* (2020) reported a 99% removal of BPA within the experimental duration of 240 minutes using both tested anode materials (Pt and BDD) and at both tested temperatures (6 and 20 °C), with a current efficiency applied at 86 mA cm^−2^, and BDD where 43 mA cm^−2^ was employed. Parastar *et al.* (2018a)^270^ reported that under the optimal conditions (10 mg L^−1^, 1500 mA, pH 7, and 0.5 cm), the removal efficiency observed varied between 60% and 90%. This research study concludes that employing the electrocoagulation process with iron electrodes under these optimal conditions can effectively remove BPA from aqueous solutions.

**Table 6:** Studies on BPA removal *via* coagulation/flocculation

Method used	Operation condition	Removal efficiency	Reference
Peroxy electrocoagulation	45 ppm, 0.1 g per L Na_2_SO_4_, 125 g per L H_2_O_2_, pH: 2, 0.11 mA cm^−2^, 45 min	80.48%	258
Photocatalysis	10 mg L^−1^, TiO_2_@MIL-101(Cr), 240 min	99.4%	259
Electrocoagulation	3.25 mg L^−1^, pH: 8.5, 12.0 mA cm^−2^, 23 min, 0.308 kW h m^−3^	98.2%	260
Photocatalytic	5.0 mg L^−1^, pH 5.0, 500 W, 0.3 g L^−1^, 180 min	100%	261
Electro-fenton	10 ppm, 10, 25 mg L^−1^, pH: 2.9, Na_2_SO_4_, Fe_2_SO_4_, 50 min	>90	262
Membrane electrochemical microfiltration	2.0 V, 50 ppm, 0.1 mol L^−1^ Na_2_SO_4,_ 0.88 min	97%	263
Microwave-Mn-fenton	100 ppm, 34.0 mg L^−1^ H_2_O_2_, 300.0 W, 6.0 min, pH: 4	99.7%	264
Iron electrode electrocoagulation	10 mg L^−1^, 25 °C, 30 V, pH = 7, 1500 mA	90%	265

### 6.2 Chemical processes

In wastewater treatment, the primary treatment mostly functions by the sorption of BPAs onto sludge *via* coagulation/flocculation and subsequent sedimentation.^271^ However, chemical treatment is also equipped to enhance the BPA removal efficiency. Some of the treatments include ultraviolet irradiation and ozonation,^222^ which are summarized in Table 7. Sulfate radical (SO_4_˙^−^)-based advanced oxidation processes (AOPs) have gained considerable interest in recent years for their capacity to effectively remove recalcitrant organic molecules, including BPA, in water and wastewater due to their strong oxidation properties. SO_4_˙^−^ has a high standard redox potential (2.5–3.1 V) at a neutral pH and shows greater sensitivity towards certain organic contaminants.^284^ SO_4_˙^−^ can be generated by activating persulfate (PS) or peroxymonosulfate (PMS) using many processes including heat, transition metals, alkaline conditions, and ultraviolet (UV) irradiation.^282^ The activation by UV irradiation was also considered to be a very successful and promising technique.^285^ The degradation rate of BPA by UV irradiation reached 270 ± 6/10^−3^ min^−1^ with 55% removal from 0.44 mmol L^−1^ initial concentration.^282^ Furthermore, the ozonation method, which involves a redox mechanism, also has several advantages including high removal efficiency, non-selectivity, and disinfection effects.^222^ BPA can be destroyed by a direct reaction with molecular ozone or indirect reaction with hydroxyl radicals, which are generated from the alkaline breakdown of ozone. Hydroxyl radicals are a more powerful oxidant with lesser selectivity, as compared to molecular ozone. Umar *et al.*^272^ reported that ozonation achieved up to 100% of BPA removal from water with the initial concentration of a maximum of 0.5 mg L^−1^. Out of all the methods, photocatalytic degradation offers an unlimited potential for BPA degradation, especially when it comes to the choice of the nanocomposite materials.^279^Table 7 shows that the photocatalytic degradation of BPA reached more than 90% efficiencies and can be achieved within a short period.

**Table 7:** Previous studies on the chemical process involved in the removal of BPA

Treatment unit	Performance removal	Reference
Ozonation	100%	272
Ozonation	17–100%	273
Ozonation	40%	274
Ozonation	33%	275
Ozonation	21.5%	276
Photocatalytic degradation (Bi_2_WO_6_/g-C_3_N_4_/BPQDs)	95.6%	277
Photocatalytic degradation (CPD-TiO_2_)	99%	278
Photocatalytic degradation (Fe–TiO_2_)	99.9%	279
Photocatalytic degradation (Fe–TiO_2_/rGO)	99.9%	279
Photocatalytic degradation (MIL-100(Fe)/PDI)	100%	280
Photodegradation	50–52%	281
Photodegradation	58%	274
UV irradiation	270 ± 6/10^−3^ min^−1^ 55%	282
UV irradiation	25–52%	283
UV irradiation	75–98%	281

### 6.3 Integrated processes

Integration treatment was used to enhance the removal of contaminants from various contaminated water or to encounter issues that arise with the single treatment. Integrated or hybrid systems were developed to meet the regulations, such as those, for law enforcement removing substances and producing drinkable water. Another advantage of hybrid systems is their ability to address the limitations of procedures by incorporating alternative methods.^286^Table 8 shows the integration treatment for the removal of BPA. The integration treatment involved a combination of physical, chemical, and biological processes and also the sequential of the single process. From Table 8, most integration studies showed a great removal of BPA. However, the integration of chemical and biological processes represents only 58.23% of removal. However, this removal percentage surpasses the treatment of 21.59% and 14.48% achieved with *Phanerochaete chrysosporium* alone and the Fenton treatment, respectively.

**Table 8:** Previous studies on the integration process involved in the removal of BPA

Integration	Process	Performance removal	Reference
Physical and chemical	Ceramic membranes incorporating manganese-cobalt oxides (MCOs) to catalyze the ozone-based degradation	90.6%	287
	Membrane filtration coupled with advanced oxidation processes (AOPs) and adsorption. Ceramic membrane filtration coupled with PMS activation and adsorption	—	288
	Ultrafiltration-catalysis membrane	95%	289
	Adsorption and catalytic ozonation: MWCNTs/Fe_3_O_4_	25 to 75 ppm (98%)	290
Physical and biological	Adsorption and biodegradation	98.1% at 5 mg per L BPA	291
	Membrane-assisted enzyme treatment	89% removal of BPA	292
Chemical and biological	Fenton process and *Phanerochaete chrysosporium*	58.23% at 1 mg per L BPA	293
	MBR and ozonation	90% removal of BPA 80 min at 8 mg O_3_ per L	294
Sequential treatment of biological reactors	An up-flow anaerobic sludge blanket (UASB), submerged aerated biological filters (SABF), and horizontal subsurface flow constructed wetland (HSSF-CW) reactors	99%	295

According to Tarafdar *et al.*, (2022), the effective strategies that can be employed to eliminate BPA are biodegradation and adsorptive processes.^296^ However, there is a need to address the issues of increased adsorption ability and rapid adsorption rates for both conventional and non-conventional adsorbents.^297^ Abu Hasan *et al.*, (2023) reviewed the proposed biological treatment and adsorption for BPA concentrations in the effluent that exceeds 1 mg L^−1^.^286^ For concentrations below 1 mg L^−1^, the utilization of an advanced oxidation process (AOP) and MF may be more appropriate, enabling load reduction and consequently decreasing operational and maintenance expenses. Godiya and Park (2022)^297^ stated that the integration of membrane separation with additional oxidation/biodegradation methods has the potential to mitigate contamination and mass transfer constraints. According to Zielińska *et al.* (2019), the integration of chemical and biological gained widespread interest in removing BPA from contaminated water.^298^ These methods primarily involve the integration of Advanced Oxidation Processes (AOPs) such as photocatalysis, ozone oxidation, Fenton and photo-Fenton oxidation, and wet air oxidation (WAO) supplementing the conventional biological treatment. Fundamentally, an effective treatment approach for the elimination of particular EDCs should involve the integration and optimization of sophisticated physical, chemical, and biological treatment techniques.^299^ It is essential to conduct an exploration and investigation of every advanced treatment process to identify any unidentified potential limitations.

## 7. Breakdown/removal mechanisms of BPA in water

The remediation process of BPA present in water can be understood from three points of view. The first involves the biodegradation mechanism, the second corresponds to the removal of mass transfer (adsorption) and the third is the degradation of the molecule by advanced oxidative processes.

### 7.1 Adsorption mechanism

**Fig. 3:**
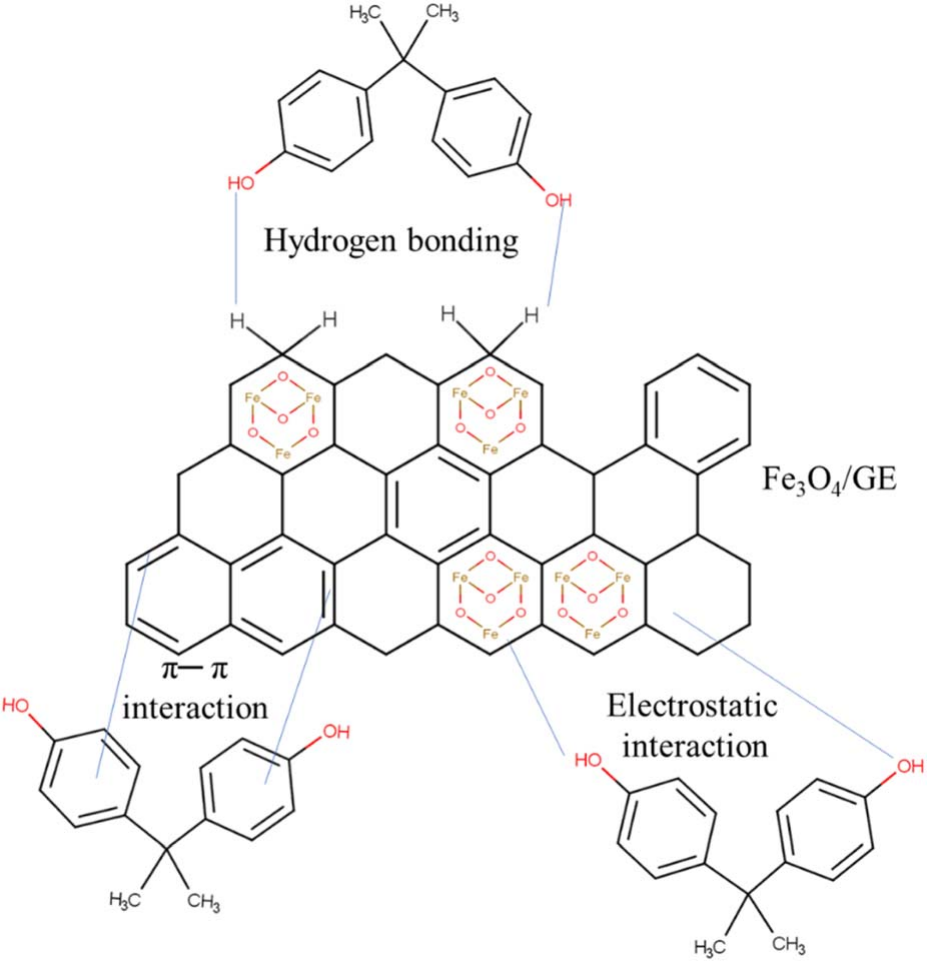
Representation of the BPA adsorption mechanism onto graphene coated with Fe_3_O_4_ nanoparticles.

The adsorption of BPA and the mechanisms that govern this process depend on some factors including the nature of the pollutant, the chemical and physical properties of the solid, and the adsorption results. The morphology and structure analyzed through characterization techniques (adsorbent) are also necessary to determine the mechanism.^300^ The coprecipitation method was used to develop magnetic nanoparticles decorated with graphene oxide nanosheets, and this material was the BPA adsorbent.^301^ The main interaction that occurred between the graphene skeleton and the benzene ring of the pollutant was π–π. In another study, graphene-coated magnetic iron oxide nanoparticles were used to remove BPA, and the fabricated aerogel (Fe_3_O_4_/GE) was subsequently reduced to ethylenediamine.^302^ The interactions were π–π, hydrogen bonding, and electrostatic, both present between the adsorbate ring and the conjugated carbon network (Fig. 3). The Fe_3_O_4_/GO adsorbent was obtained by the hydrothermal method where the BPA ring interacted with the surface *via* π–π interactions, and sp^3^ hybridization also corroborated the remediation process.^303^ In summary, we can see that the nature of the interactions can be widely varying. However, BPA does not breakdown *per se* but is attached to the adsorbent surface *via* these physico-chemical interactions and thus removed.

### 7.2 Membrane/nano-filtration mechanism

Interactions such as hydrophobicity, hydrogen bonding, and electrostatic interactions are essential in the remediation of BPA in membrane filters or even nanofilters. One study used a synthetic effluent with BPA, which was remediated in a submerged membrane bioreactor.^304^ The researchers reported that almost complete removal (99%) was obtained at a low volumetric loading rate (0–21.6 g per m^3^ per day). Biodegradation and adsorption mechanisms were responsible for the efficient result for the membrane use. The adsorption process showed endothermic behavior favorable to high temperatures and hydrophobic character, with van der Waals and electrostatic interactions. These were present between the BPA molecules and the reactor sludge. Electrospinning was used to obtain a 6,6-nanofibrous membrane for BPA remediation.^305^ The maximum capacity of 91 mg g^−1^ was obtained *via* hydrogen interactions between the adsorbate hydroxyl and the membrane carbonyl and hydrophobic interactions. BPA is nanoscale in size, so size exclusion through the nanofibrous membrane could not support any removal. In another study, a nanofibrous membrane carrying the laccase enzyme was synthesized on polyethersulfone and polyethersulfone membranes to remove BPA.^306^ The hydrophilic nature of the laccase membrane enabled 90% removal, being governed by hydrogen bonds of the amine group present in the membrane with BPA. Hydrophobic interaction between polyethyleneimine/polyethersulfone and the adsorbate and electrostatic interaction with the cationic group of the polyethyleneimine/polyethersulfone and the pollutant molecule. The mechanism of reverse osmosis and nanofiltration membranes was analyzed concerning BPA removal.^307^ The authors highlight that membranes made of polyamide present greater rejection of BPA when compared to those made of cellulose acetate. The mechanisms correspond to hydrogen bonding, electrostatic, and hydrophobic interaction (Fig. 3).

### 7.3 Photocatalytic degradation and its mechanism

**Fig. 4:**
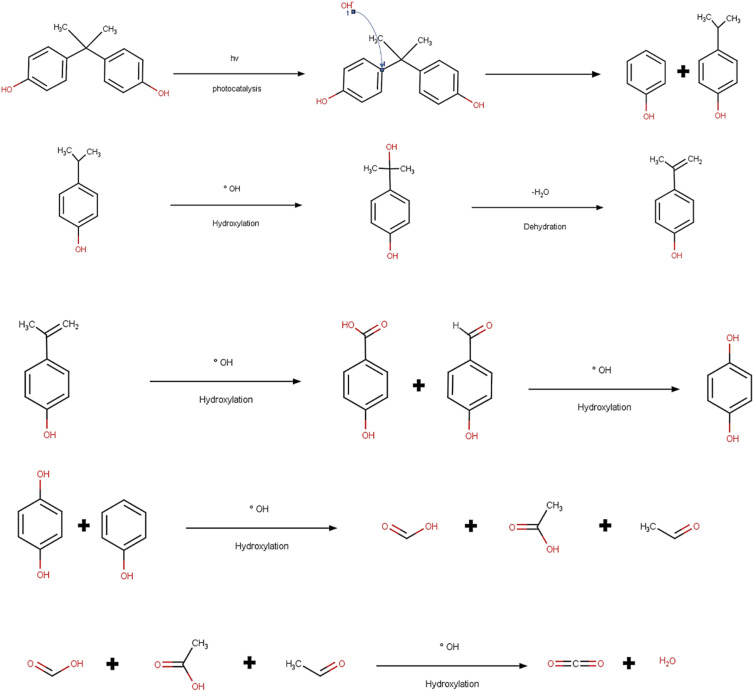
Schematic representation of the photocatalytic degradation process of BPA.

The photodegradation of BPA involves mechanisms that correspond to the generation of electron holes present in the conduction band and also the electrons contained in the valence zone after the irradiation step.^308^ Studies have concluded that depending on the type of photocatalyst, the oxidation of the valence band and hydroxyls are mainly responsible for the degradation.^309^ Therefore, the hydroxyl initiates the degradation by directly attacking the carbon present in the phenyl group, and then 4-isopropylphenyl and hydroquinone are generated as intermediates. In the case of 4-isopropylphenyl, it is again converted to 4-(2-hydroxypropan-2-yl) phenol, and then, after hydroxylation and dehydration, it generates 4-hydroxybenzoic acid and 4-hydroxybenzaldehyde (Fig. 4). Researchers describe that the products can also undergo oxidation producing various organic acids that are also degraded, finally forming products that are not toxic such as water and CO_2_.^310,311^

### 7.4 Advanced oxidation processes and its mechanism

**Fig. 5:**
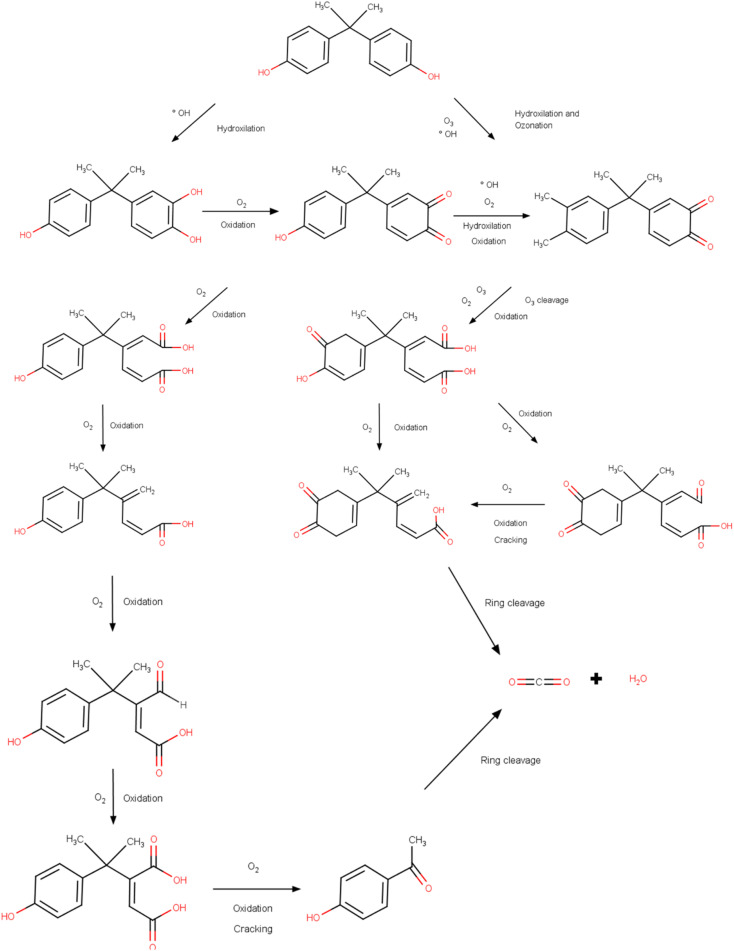
BPA decomposition mechanism in the presence of Fe_3_O_4_–MnO_2_ and ozone.

Due to its properties, ozone is highly oxidizing and is present in the oxidation of several organic compounds, such as BPA. The reactions can be indirect or direct. The oxidation process of BPA and molecular ozone occurs in a basic environment through an indirect reaction, while in an acidic environment, decomposition and oxidation occur through the elimination of radicals in a direct reaction. Molecular ozone also attacks structures rich in electrons (non-protonated amines, activated aromatic compounds, and unsaturated bonds such as C=C). The presence of phenolic groups increases the chances of a reaction. In this sense, degradation *via* ozonation obtained complete decomposition and 30% mineralization of BPA under acidic conditions.^312,313^ After 25 min, the stoichiometric ratio between ozone and BPA was 10.3. The production of ozonoids and the attack of ozone on C=C were the main mechanisms involved. Malonic and oxalic acids accelerate the degradation of ozonides, corroborating with simultaneous mineralization. A nanocomposite (AC/CeO_2_/ZnO) was synthesized to remove BPA.^314^ Under optimal conditions, total carbon removal was observed (time of 60 min, pH of 8, and 500 μg per L catalyst dose) with the use of five degradation products. OH radicals were dominant in the process and increased under basic conditions. The generation of organic acids with the addition of OH reduced the pH. Under pH conditions of 11, BPA removal was 100% using pumice that deposited 4 g L^−1^ of O_3_/*n*TiO_2_/H_2_O_2_ and 12 g L^−1^ of O_3_/*n*ZrO_2_/H_2_O_2_ in 30 min of contact.^315^ The increase in peroxide at first increased the generation of OH, improving BPA removal; in a second moment, the efficiency reduced due to the generation of fewer oxidizing peroxide radicals. The catalytic degradation of BPA was analyzed with the Fe_3_O_4_–MnO_2_ catalyst *via* ozonation.^316^ Under conditions close to neutrality and 100 mL min^−1^ flow rate, approximately 97% of BPA was removed. Regarding the specific mechanism, it should be noted that different mechanisms were proposed. Zhang *et al.* (2020) proposed that the ozonation process leads to the decomposition of the BPA (Fig. 5) and generates 11 intermediates before the ring cleavage and generation of carbon dioxide and water.^316^

### 7.5 Electrochemical degradation and its mechanism

**Fig. 6:**
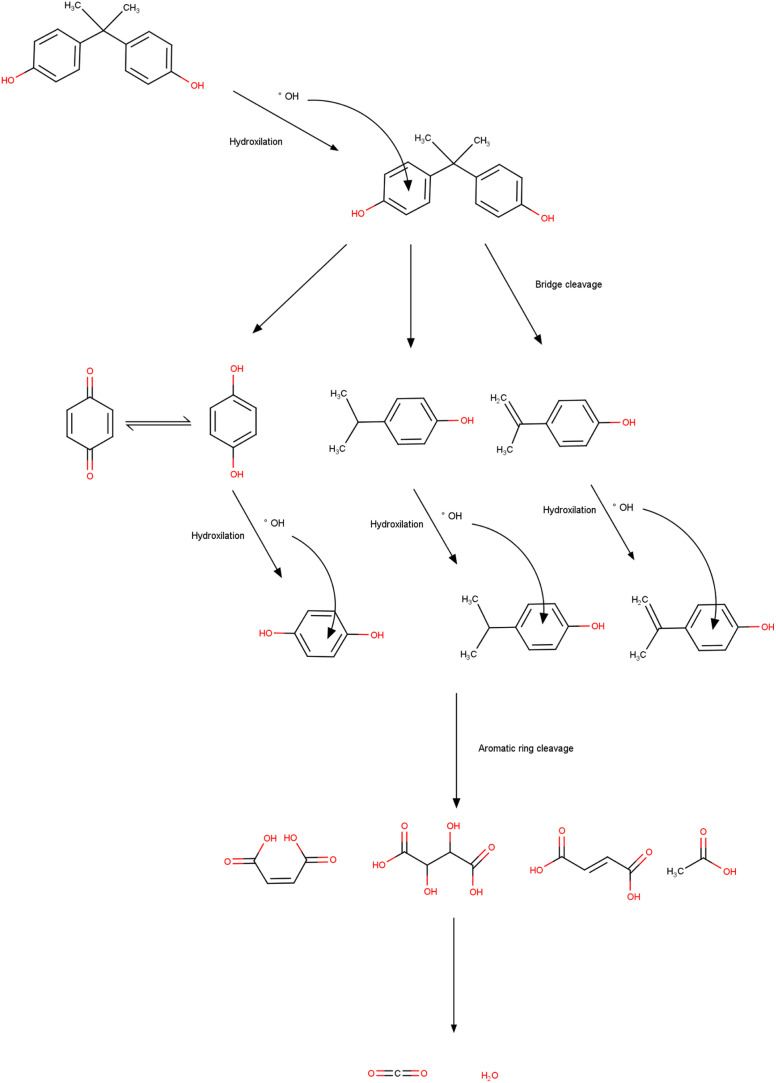
BPA electrochemical degradation routes.

In a pulsed plasma discharge system containing activated carbon, 95% was obtained through electrochemical degradation in one hour of reaction and with a 20 kV discharge.^317^ In the presence of oxygen and acidic conditions, the degradation was superior. First, the process began with attacks of the hydroxyls and the cleavage of the isopropylidene bridge. The electrophilic substitution reaction by O* led to the oxidation of the pollutant, and then the products generated by the oxidation were transformed into single-ring aromatic compounds. Then, the cleavage of the ring generated oxalic, formic, and acetic acids, and finally, they were mineralized into carbon dioxide and water. Graphite/β-PbO_2_ anodes and granular activated carbons were used to remove BPA.^318^ The maximum efficiency of 99% was obtained *via* two mechanisms that use OH. One of them starts with the loss of the hydroxyl group and a ring-opening reaction leading to the final production of acetic acid and ethane. In the second pathway, the beginning occurs by the oxidation of 4-(2-hydroxypropan-2-yl) phenol with the sequence of ring opening producing carbon dioxide and water at the end. An experiment also studied electrochemical peroxidation to remediate BPA present in groundwater.^319^ The BPA electrochemical degradation can be summarized as given in Fig. 6. It starts with the BPA hydroxylation, due to the OH radicals present. Due to the instability of the new intermediate, cleavage occurs at the isopropylidene bridge, generating one-ring compounds. These new aromatic groups undergo the cleavage process, which are further mineralized onto CO_2_ and H_2_O.^320,321^

### 7.6 Photo-fenton process and its mechanism

**Fig. 7:**
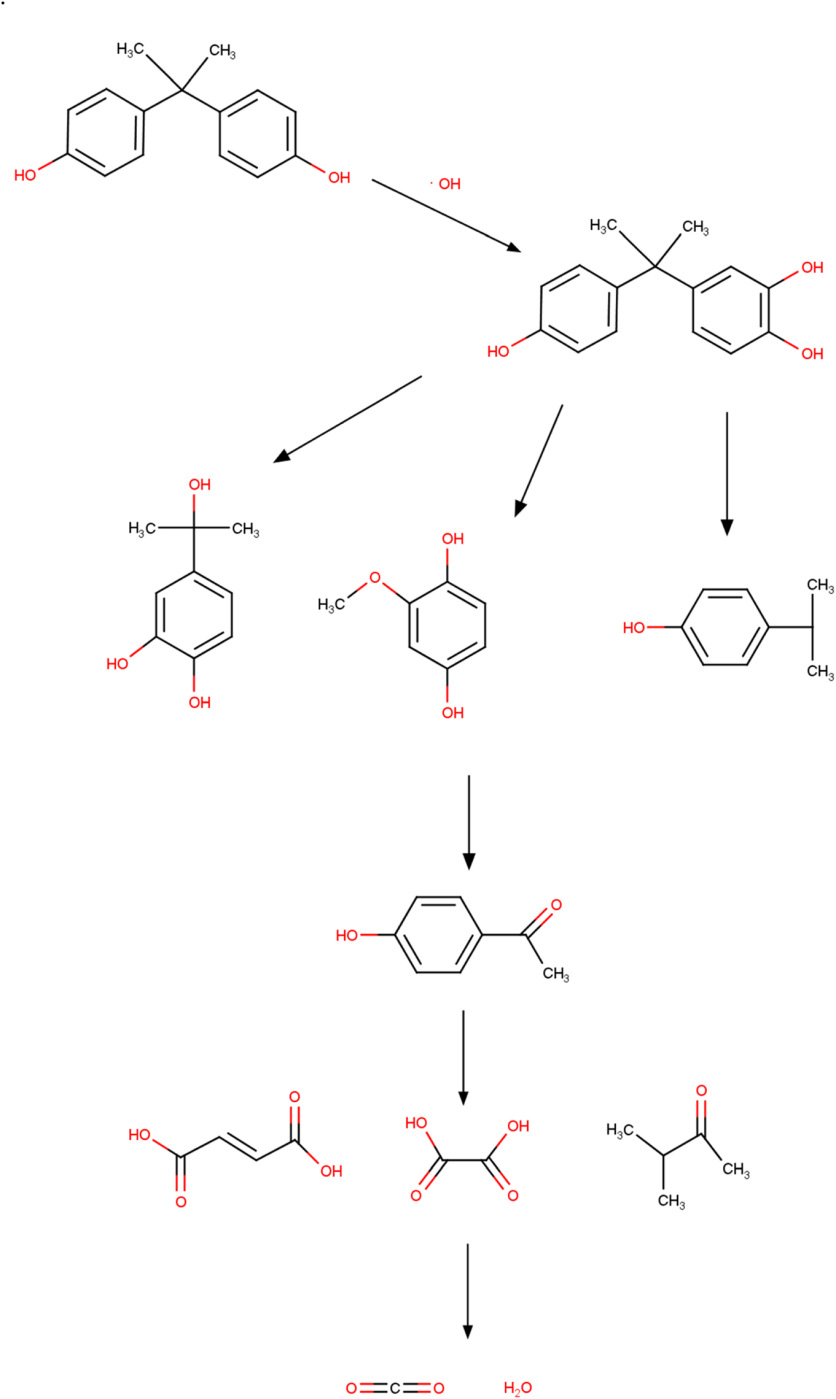
BPA degradation mechanism for the photo-Fenton reaction.

The BPA degradation mechanism through the photo-fenton process has been reported by different authors. da Silveira Salla *et al.*^322^ have proposed the mechanism of BPA in the presence of CuFeS_2_ and visible light, as given in Fig. 7. First, the BPA is converted into hydrolyxated bisphenol, due to the ˙OH radicals. After that the ˙OH radicals tend to interact once again by promoting the BPA bridge cleavage. Leading to the formation of 4-isopropylphenol, 4-hydroxyacetophenone, 2-methoxyhydroquinone, and 4-(1-hydroxy-1-methylethyl)benzene-1,2-diol. The later intermediates suffer from another hydroxylation, which leads to the ring opening and the formation of acids in the form of oxalic, fumaric, and isobutryic acids. Last, the acids are mineralized forming CO_2_ and H_2_O. Other differences generated on the mechanism pathway can be attributed to the catalyst employed. Zhu *et al.* (2018) indicate that the catalyst based on Ag/AgCl nanoparticles onto ferrite can produce electron–hole pairs due to the surface plasmon resonance.^29^ These electron pairs are further transferred to the ferrite, which increases the conversion of Fe^3+^ into Fe^2+^. The latter also affects the number of radicals produced due to Fe^2+^ reacting with the peroxide (H_2_O_2_). The same mechanism was also confirmed by Liu *et al.*^323^ when they employed Ag/AgCl/Ferrite-S, indicating that the electron–hole pair generation migrates to the conduction band before acting in the Fe^3+^-to-Fe^2+^ conversion mechanism.

### 7.7 Biodegradation and its mechanism

Following the other mechanism trends, the degradation of BPA has similar routes regarding the hydroxylation and bridge/aromatic cleavage; however, new routes tend to emerge due to the microorganism present. The removal of BPA in biodegradation mechanisms involves aerobic and anaerobic conditions, together with the action of microorganisms. The use of *Bacillus* sp. cultivated in anaerobic and aerobic media was applied in the remediation of BPA.^324^ In this study, the strain that was extracted obtained the best performance with approximately 51% mineralization using 5 mg L^−1^ of pollutant concentration. The aerobic process was the most efficient, generating approximately 5 final products. The entire process was dominated by electron acceptors such as Fe^3+^, SO_4_^2−^, and NO_3_ for the anaerobic system, while the acceptors of the aerobic system were O_2_. The acceptors SO_4_^2−^ and NO_3_ did not influence biodegradation; however, Fe^3+^ was the most efficient when added to the anaerobic system. Despite the good performance, the rate was not higher than that obtained in the system containing oxygen. In another study, the strain YC-JY1 of *Sphingobium* sp. was obtained to remove BPA; the results show that at a pH of 6.5, degradation was maximum.^325^

**Fig. 8:**
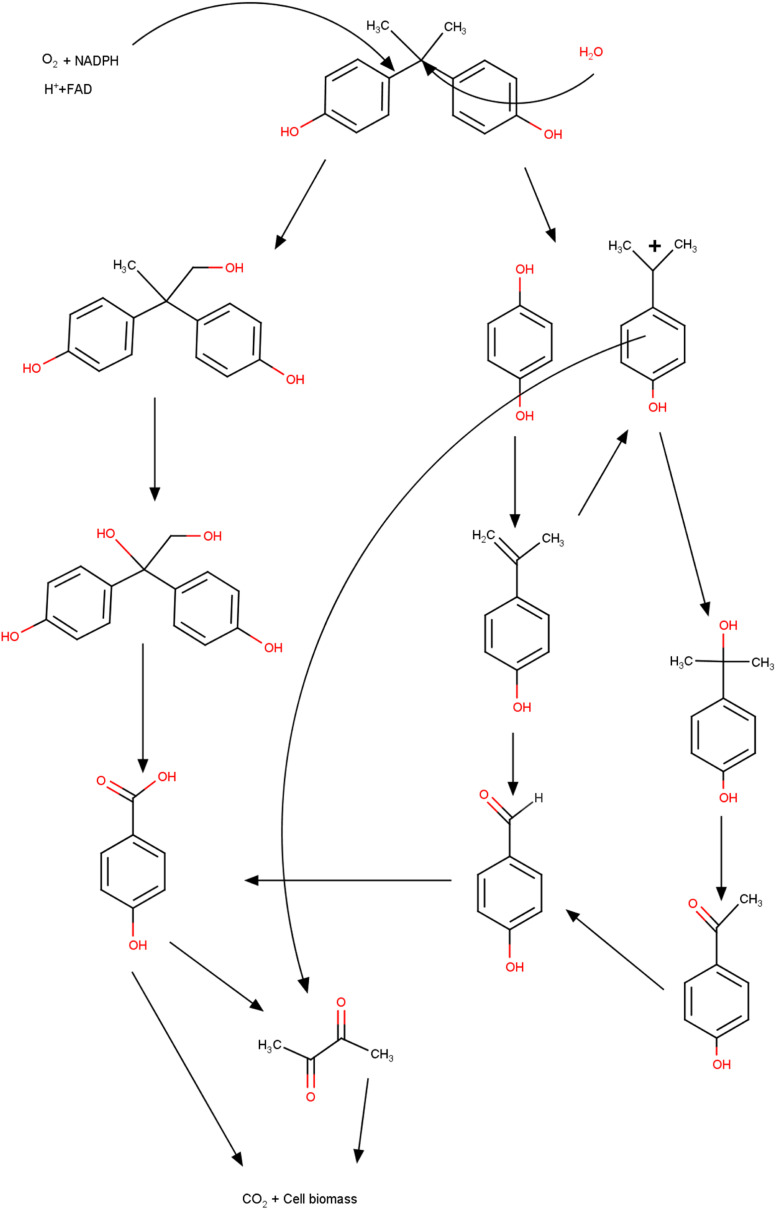
BPA biodegradation routes with *Sphingomonas* sp. present in the solution.

Golshan *et al.*^326^ investigated the BPA degradation mechanism in the presence of several microorganisms. From all the investigated microorganisms, the author evaluated *Sphingomonas* sp. as given in Fig. 8. At a temperature of 30 °C, the process involved two mechanisms, the first of which corresponds to the conversion of the pollutant into 1,2-bis(4-hydroxyphenyl)-2-propanol, which is then transformed into 4-hydroxybenzaldehyde and 4′hydroxyacetophenone, for final use by the bacteria, and the second mechanism corresponds to the conversion of BPA, sequentially generating the accumulation of conversion products (process medium). These products correspond to the same ones obtained in the degradation and photocatalytic biotransformation of BPA described previously. The addition of salt-tolerant species corresponds to the biological treatment carried out with the concentration of 50 mg L^−1^ of pollutant, contained in a sequential batch reactor with saline wastewater.^326^

Similarly, two biodegradation processes that correspond were also found by Wang *et al.*^327^ The first is the cleavage of the C=C bond and hydroxylation. In the latter, BPA is first transformed into 2,3-bis(4-hydroxyphenyl)-1,2-propanediol and 2,2-bis(4-hydroxyphenyl)-1-propanol, which are mineralized in cell biomass and carbon dioxide. The second corresponds to the cleavage step of the C=C bond, where degradation begins in the benzene ring and oxidative cleavage, and aliphatic acids are subsequently generated, which are mineralized in cell biomass and carbon dioxide. In another study, green algae *Desmodesmus* sp. was used to detoxify and biodegrade BPA.^327^ The mechanisms encompass three dynamics. The first corresponds to the oxidative cleavage of the pollutant to monophenols, followed by their mineralization. The second corresponds to hydroxylation generating monohydroxybisphenol-A, followed by hydroxymethylation forming 2-hydroxy-3-hydroxymethylbisphenol-A. The third is glycosylation (a biochemical process that consists of the addition of sugars to proteins, lipids, or other organic compounds, resulting in the formation of glycoconjugates).

## 8. Challenges and future directions

When searching the phrase ‘BPA’, it is observed that it is a highly studied compound and that it presents a large number of studies, ranging from its presence in the environment, entry routes, and toxic potential in animals, humans, aquatic life and plants, to biological, physical, and chemical processes for their environmental mitigation. All of these efforts and interests are related to their constant presence and release into the environment, due to the means of production and the contemporary culture of consumerism. When analyzing toxic effects, a vast field is observed, where different pathways in the human body can be affected, where it presents a strong correlation with other factors, such as concentration, exposure time, and route of contact. Another aspect is the age at which humans come into contact with BPA. In this sense, it is clear that babies and children in the development phase are the most affected.

Based on this, the implicit mechanisms require better understanding and more investigations, this is one of the challenges that BPA remediation processes face. More reliable technologies for detecting and measuring plastic exposure are also needed. The complexity lies in the different routes of exposure due to its presence in various products, so it can be through dermal contact or oral or olfactory ingestion. Experiencing the availability of highly accurate methods of measuring exposure makes it possible to estimate the level of danger and determine the best interventions to achieve high efficiency in reducing damage. More studies are also needed to analyze and describe the damage along the food chain and prolonged exposure to low concentrations of BPA. Although it is possible to observe studies that show that low concentrations do not present a health risk, other researchers highlight the cumulative potential of BPA over years of exposure. Only by understanding long-term damage and at low concentrations is it possible to establish safe guidelines and regulations by authorities through competent bodies. Among the countless challenges to be overcome, there is also the field that seeks to develop new, more sustainable materials that can replace BPA in the most diverse consumer products. The points observed by the researchers are economic viability and health risk, an example being vegetable polymers. Even if BPA were completely replaced, its presence in the environment would persist for a long time; therefore, more studies are needed to describe its impacts at the aquatic and plant level. These studies must go hand in hand with new BPA mitigation strategies.

Bacterial degradation has been shown to be 45–100% efficient depending on the strain of bacteria and the choice of process parameters. To obtain total optimization of the biodegradation process using bacteria, it is necessary to carry out continuous investigations in different regions of the world, as meteorological variables are different and have a high influence on colony growth. The seasons must also be taken into account as well as the presence of other chemical molecules in addition to BPA. Regarding other remediation strategies, BPA can be removed from water by physical processes such as membrane filtration, adsorption, and coagulation and by chemical processes such as ozonation and photocatalytic degradation. We see that membrane processes can remove 40–95% BPA in water with a high level of throughput. Other physical processes such as adsorption and coagulation can remove up to 100% BPA in water but throughput is significantly lesser. Chemical processes such as ozonation and photocatalytic degradation have achieved similar performance levels. However, we believe the use of hybrid/integrated processes can simultaneously achieve high removal efficiency at high water throughput due to the intensification of the process. It is encouraged that researchers explore various hybrid technologies for BPA removal from water with the aim of achieving process efficiency and scalability.

## 9. Conclusion

A detailed analysis of the literature was conducted to determine the toxicity levels and concentration of BPA in environment-quality plastic packaging. The degradation process using different species of bacteria made it possible to describe the damage to humans, animals, and plants that BPA can cause through prolonged exposure, and the role that these organisms play. It was observed that food-grade packaging contains considerable concentrations of BPA, a utilization route that had inadvertently led to biomagnification and its ubiquitous nature in the aqueous environment. This has increased the need for remediation techniques to reduce its incidence in the aqueous environment. Researchers should focus their efforts on providing a better understanding of the mechanisms of the degradation of BPA and its toxic effects on the ecosystem. It was observed that physical processes are able to achieve >90% removal efficiency of BPA from water but tend to perform better under a low pollutant load in the influent stream. Chemical processes show wider variation in performance with 20–100% removal efficiency, while integrated processes show 90–100% removal efficiency of BPA from water. It is highly necessary to obtain new routes to replace BPA in various consumer products, without it losing its quality. Therefore, it is highly important to explore new solutions by developing efficient and viable strategies for plastic waste management. In soil, BPA can be effectively remediated by bacterial degradation. Aqueous phase remediation of BPA can be achieved with processes such as membrane filtration, adsorption, coagulation, ozonation, and photocatalytic degradation. It was proposed that hybrid/integrated processes are a strategy that can help achieve high removal efficiency at high wastewater throughput.

## Ethical statement

This article does not contain any studies involving human or animal subjects.

## Data availability

No primary research results, software or code has been included and no new data were generated or analysed as part of this review.

## Conflicts of interest

The authors declare that there are no conflicts of interest.
